# Assessment of Corrosion Product Formation (Iron Oxides and Oxyhydroxides) on Carbon Steel in Saline Media Containing Protic Ionic Liquids by Advanced Structural and Microstructural Characterization

**DOI:** 10.1021/acsomega.6c01592

**Published:** 2026-06-03

**Authors:** Caio Victor Pereira Pascoal, Daniel de Castro Girão, Bruno Gomes Linhares, Samuel Lucas Santos Medeiros, Regiane Silva Pinheiro, Seyed Ali Razavi, Mohammad Rezayat, Igor Frota de Vasconcelos, Hosiberto Batista de Sant’Ana, Gemma Fargas, Walney Silva Araújo

**Affiliations:** † Department of Metallurgical and Materials Engineering, Federal University of Ceará, 60440-900 Fortaleza, CE, Brazil; ‡ Department of Food Engineering Federal University of Maranhão (UFMA), Imperatriz, Ma 65915-060, Brazil; § Department of Chemical Engineering, Federal University of Ceará Fortaleza (UFC), Fortaleza, CE 60440-554, Brazil; ∥ Center for Structural Integrity, Micromechanics, and Reliability of Materials (CIEFMA)-Department of Materials Science and Engineering, Universitat Politècnica de Catalunya-Barcelona TECH, 08019 Barcelona, Spain; ⊥ Department of Materials Science and Engineering, EEBE, Universitat Politècnica de Catalunya, UPC, C/Eduard Maristany, 10-14, 08019 Barcelona, Spain; # Barcelona Research Center in Multiscale Science and Engineering, 16767 Universitat Politècnica deCatalunya, UPC, C/Eduard Maristany, 10-14, 08019 Barcelona, Spain

## Abstract

This study investigates protic ionic liquids (PILs) as corrosion inhibitors for carbon steel in a saline environment, focusing on their influence on the formation and stability of corrosion products during exposure. By examining corrosion product evolution under inhibited conditions, this work provides a mechanistic perspective beyond conventional inhibition efficiency, evaluating whether the resulting corrosion layers can act as a protective barrier during prolonged exposure. X-ray diffraction, Mossbauer, and Raman spectroscopy techniques were applied to characterize the surface and corrosion product. Moreover, optical and scanning electron microscopy techniques (SEM–ED and FIB–SEM) were selected to identify the oxides/oxyhydroxides externally. At the same time, X-ray spectrometers with energy dispersion were used to analyze the elemental composition, mainly O, Fe, N, Cl, and Na. Fundamentally, this preliminary investigation seeks to identify corrosion products (iron oxides/oxyhydroxides), including lepidocrocite, magnetite, and goethite, elucidate their formation mechanisms, and contribute a novel perspective to the corrosion inhibitor literature. In particular, the study focuses on the interaction between carbon steel and Protic Ionic Liquids (PILs), exploring their potential influence on the formation and evolution of distinct oxide phases on the material’s surface. As a key distinguishing feature, the addition of the corrosion inhibitors PILs (2-HEAF) and (2-HDEAF) revealed the presence of a dense and compact goethite, a phase that can aid in blocking chloride penetration, potentially extending the longevity of carbon steel in saline environments. This observation supports the efficacy of these inhibitors, as previously demonstrated in electrochemical and weight-loss experiments, suggesting their potential for long-term corrosion protection.

## Graphical abstract

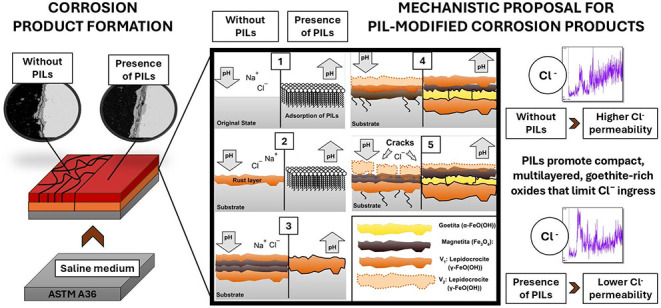

## 1 Introduction

Corrosion is a significant global issue, with each country losing 3–5% of its GDP annually due to actions taken to combat it.^1,2^ The National Association of Corrosion Engineers (NACE) estimated global economic losses due to corrosion at approximately 2.5 trillion dollars in 2016.^3–5^ Among the various methods to combat corrosion, inhibitors are widely used because they are practical and effective under various pH values, temperatures, and pressures. However, some inhibitors pose risks to both humans and the environment.^6–9^ The ASTM A36 is a carbon steel widely used in the chemical and oil industries due to its high structural quality and low application cost. However, this material, being a low-alloy carbon steel, presents major problems associated with marine environment corrosion. Therefore, to evaluate this material in its primary form, this work presents an innovative and initial evaluation of the correlations between physical characteristics and anticorrosive properties when protic ionic liquids are applied as corrosion inhibitors in low-carbon steel samples (ASTM A36) in NaCl 3.5% wt. solution.^10,11^

A key challenge in improving corrosion resistance is the limited understanding of the chemical characterization of the oxide/oxyhydroxide layers formed on metals and their formation mechanisms^12,13^ This understanding is crucial for advancing the development of materials with enhanced corrosion resistance. Although ionic liquids, particularly aprotic ones, are considered promising corrosion inhibitors due to their high ionic character and excellent thermal stability,^14–17^ some are problematic because of their tendency to bioaccumulate in organisms and soil, raising environmental concerns.^18,19^

In contrast, protic ionic liquids (PILs) have emerged as promising and more environmentally friendly alternatives for corrosion inhibition.^20–24^ However, they remain comparatively underexplored, particularly regarding their influence on the formation, characterization, and protective role of oxide layers on carbon steel.^25^ Additionally, the research field that most extensively investigates the formation of oxides and oxyhydroxides on carbon steel in saline environments is typically atmospheric corrosion.^26–28^ However, comparatively limited attention has been given to studies evaluating oxide formation in electrolytic solutions containing corrosion inhibitors. Unlike many conventional organic inhibitors, which may present issues related to volatility, toxicity, or environmental persistence, PILs are formed through a simple proton transfer between a Brønsted acid and a Brønsted base, resulting in compounds with negligible vapor pressure, good thermal stability, and tunable physicochemical properties.^29,30^

These features allow the molecular structure of PILs to be tailored to improve their interaction with metallic surfaces.^31^ In addition, the ionic character of PILs can promote stronger adsorption of the metal and solution interface through electrostatic interactions, hydrogen bonding, and coordination with active surface sites. Such interactions may not only form an adsorbed protective layer but also influence the formation and stability of corrosion products, potentially leading to more compact and protective oxide or oxyhydroxide layers.^32^ Despite these advantages, studies addressing the role of PILs in controlling corrosion product formation and surface chemistry on carbon steel are still limited. Therefore, investigating these interfacial processes is essential to better understand their potential as sustainable corrosion inhibitors.^21,33–35^

The corrosion properties of carbon and weathering steels exposed to different atmospheric conditions have been widely investigated using techniques such as X-ray diffraction and Raman microscopy.^28,36^ These studies consistently identify lepidocrocite (γ-FeOOH),^37,38^ goethite (α-FeOOH),^37,38^ and magnetite (Fe_3_O_4_)^39^ as the primary corrosion phases formed on the steel surface. In addition to phase identification, several researchers have examined the morphology of the oxide layers developed on carbon and weathering steels, reporting that these predominant phases exhibit distinct and characteristic structural features.^20,40^

In this context, lepidocrocite typically forms during the initial stages of atmospheric corrosion and, with prolonged exposure, may progressively transform into goethite, reflecting the maturation of the corrosion product layer.^20,40–44^ Morphologically, lepidocrocite is commonly observed as small crystalline globules, often referred to as sandy crystals, or as fine plate-like structures with a flowery appearance.^45^ As the corrosion process evolves, goethite may develop either as spherical, cotton-ball-like aggregates associated with semicrystalline forms interconnected by nest-like structures, or as well-defined acicular morphologies characteristic of more crystalline phases. Magnetite, when present, is generally identified as dark, compact, and relatively flat regions, frequently exhibiting circular disk-like features.^46,47^ In this study, the accumulation of protic ionic liquids (PILs) was investigated as a significant factor affecting the formation of corrosion products, specifically oxides and oxyhydroxides, on carbon steel (ASTM A36). The fundamental objective of this study was to determine whether the accumulation of PILs correlates with the formation of specific oxide phases capable of developing a compact and mechanically stable protective film, thereby potentially enhancing corrosion resistance over extended exposure periods.

In detail, A 3.5 wt % NaCl solution was used as the corrosive medium and maintained constant throughout all experiments, both in the absence and presence of protic ionic liquids (PILs). This electrolyte is widely employed as a representative saline environment because it simulates chloride-rich conditions commonly encountered in marine and industrial systems. By keeping the chloride concentration constant, the study isolates the effect of PILs on the corrosion process, enabling the formation and evolution of oxide and oxyhydroxide phases to be interpreted mainly as a consequence of inhibitor–surface interactions.

Beyond their electrochemical inhibition performance, PILs may influence the nucleation, growth, and stability of corrosion products formed on the steel surface. The adsorption of protonated ionic species at the metal and solution interface can modify local electrochemical conditions, thereby affecting iron dissolution and oxide precipitation kinetics. As a result, specific corrosion phases may be preferentially stabilized under inhibited conditions. The formation of compact oxyhydroxide phases such as Goethite (α-FeOOH) may be favored, which is often associated with improved protective behavior due to its lower porosity and higher structural stability compared with metastable phases such as Lepidocrocite. Consequently, evaluating the relationship between PIL adsorption and corrosion product formation provides important insights into the mechanisms responsible for the enhanced corrosion resistance observed in inhibited systems.

A comprehensive set of techniques was employed to evaluate the effects of PILs on a saline solution on a steel surface. In detail, Nuclear magnetic resonance (NMR) was used to characterize the PILs, while X-ray diffraction (XRD) and Raman spectroscopy were applied to analyze the oxides formed. In addition, optical microscopy (OM), and scanning electron microscopy (SEM), coupled with energy-dispersive X-ray spectroscopy (EDS), were used to identify microscopically and evaluate the corrosion products. Finally, microhardness testing was performed to quantify the oxides present on the metal surface. This study aims to evaluate the influence of protic ionic liquids on the formation of corrosion products on carbon steel, with particular emphasis on oxides and oxyhydroxides. Specifically, it seeks to establish whether the presence of PILs correlates with the development of protective oxide layers capable of limiting chloride ion permeability and providing enhanced corrosion resistance over extended periods. By addressing the need for sustainable corrosion inhibitors, this work contributes to a deeper understanding of how PILs affect oxide formation on carbon steel, helping to fill a gap in the literature regarding the protective capacity of these oxides in saline environments.

## 2 Experimental Section

### 2.1 Protic Ionic Liquid Synthesis and Characterization

The synthesis and chemical composition of the ionic liquids used as corrosion inhibitors in this study have been previously reported in detail by the authors. Readers are referred to these publications for a comprehensive description of the preparation procedures and physicochemical characterization of the ionic liquids. In the present work, the protic ionic liquids (PILs) were predominantly used at a concentration of 1000 ppm, corresponding to the optimized condition previously established in earlier studies performed by the authors under similar saline environments.

Those investigations primarily focused on the electrochemical performance and inhibition efficiency of the PILs; therefore, the optimized concentration was adopted here to enable a more focused investigation of corrosion product formation and surface-related processes on carbon steel.^21^ To further evaluate whether the inhibitor concentration could influence the formation and composition of corrosion products, additional experiments were carried out at concentrations of 500 and 1000 ppm for the X-ray diffraction (XRD) and Raman spectroscopy analyses.

This comparison was included to assess potential differences in oxide phase formation and structural characteristics of the corrosion products formed on the steel surface. The selected concentration range is also consistent with values commonly reported for organic corrosion inhibitors in saline media, supporting the relevance of the experimental conditions to practical corrosion mitigation scenarios.

In summary, 2-hydroxy diethanolamine formate (PIL 01 = 2-HEAF), 2-hydroxy ethanolamine formate (PIL 02 = 2-HDEAF), 2-hydroxy ethanolamine propionate (PIL 03 = 2-HEAP), 2-hydroxy diethanolamine propionate (PIL 04 = 2-HDEAP), 2-hydroxy ethanolamine pentanoate (PIL 05 = 2-HEAPe) and 2-hydroxy diethanolamine pentanoate (PIL 06 = 2-HDEAPe) were used as chemical nomenclature.^48–51^

The reagents were purchased from Aldrich with mass purity of 0.99 and the carboxylic acids from Sigma (mass purity of 0.996) to ensure the quality and reliability of the final products, details Table 1.^48,50–53^

**Table 1:** Classification of Protic Ionic Liquids

Abbreviations	(PILs)	Nomenclature
2-HEAF	PIL 01	2-Hydroxy ethyl ammonium formate
2-HDEAF	PIL 02	2-Hydroxy diethyl ammonium formate
2-HEAP	PIL 03	2-Hydroxy ethyl ammonium propionate
2-HDEAP	PIL 04	2-Hydroxy diethyl ammonium propionate
2-HEAPe	PIL 05	2-Hydroxy ethyl ammonium pentanoate
2-HDEAPe	PIL 06	2-Hydroxy diethyl ammonium pentanoate

### 2.2 Sample Preparation for General Evaluations

The chemical composition of the structural steel spent in the experiments (wt %) was determined with structural steel A36 (ASTM) using PDA 7000 Optical Emission Spectrometer (Shimadzu/Japan), with average: C = 0.21029%, Si = 0.03306%, Mn = 0.50905%, P = 0.00569%, S = 0.00841%, Ni = 0.02425%, Cr = 0.0233%, and Fe = 98.71%, similar composition to the literature data.^54,55^ First, the steel samples (ASTM A36) with a size of one cm^2^ were ground with 120, 220, 400, 600, and 1200 grit every paper without further polishing. Before electrochemical evaluation, the material was washed with distilled water and ethanol.^56^

### 2.3 Corrosion Evaluation by Immersion Measurements

This study aimed to elucidate the effect of the external environment on the formation of corrosion product layers. ASTM A36 carbon steel coupons (1.0 × 3.5 × 0.5 cm) were prepared using a standardized surface treatment consisting of mechanical abrasion, rinsing, and drying prior to immersion. Immersion experiments were conducted in 100 mL of a 3.5 wt % sodium chloride (NaCl) solution, with the volume defined based on the total exposed surface area.^21,34,57^ Tests were performed in the absence and presence of protic ionic liquids (PILs 01-06) at concentrations of 500 and 1000 ppm for an exposure period of 24 h. Significantly, the additional analyses focused on samples treated with PIL 01 (2-HEAF) and PIL 02 (2-HDEAF), called in this manuscript as corrosion inhibitors by (CI),which exhibited superior inhibitor performance in previous electrochemical and gravimetric evaluations. It is important to emphasize that all corrosion experiments were conducted under strictly controlled electrolyte conditions to ensure that any differences observed in corrosion product formation could be attributed primarily to the presence of the protic ionic liquids rather than to variations in the corrosive environment. In this study, a 3.5 wt % NaCl solution was used as the electrolyte for all immersion tests, maintaining identical chloride ion concentrations for both inhibited and uninhibited systems. This approach allows the evaluation of the specific influence of the PIL molecules on the corrosion process, particularly regarding their interaction with the metal surface and their potential role in modifying the nucleation and growth of oxide and oxyhydroxide phases.

Since chloride ions are known to strongly influence the formation of corrosion products on carbon steel, maintaining their concentration constant across all experiments ensures that the observed differences in phase composition, morphology, and layer compactness are associated with inhibitor–surface interactions rather than changes in environmental chemistry.

In addition, the selected immersion period of 24 h was sufficient to allow the development of a measurable corrosion product layer while still representing an early stage of corrosion evolution. Under these conditions, the influence of inhibitors on the formation and stabilization of specific oxide phases can be more clearly observed. Previous studies have shown that organic inhibitors may alter the local electrochemical environment near the metal surface by adsorbing onto active sites, thereby reducing dissolution kinetics and influencing the precipitation of corrosion products. Consequently, the formation of denser and more stable oxyhydroxide phases, such as goethite, may be favored in inhibited systems, whereas uninhibited conditions often lead to the predominance of more porous phases such as lepidocrocite. These differences in corrosion product structure play a critical role in determining the protective behavior of the corrosion layer.

### 2.4 Corrosion Morphology Analysis

The carbon steel specimens exposed to the blank (3.5 wt % NaCl) and inhibitors (PILs) with 500 and 1000 ppm concentrations for 24 h at 298 K were selected for the morphological studies. At first, to evaluate the samples after exposition, the optical microscope (OM) OLYMPUS model BX-51 M (Tokyo, Japan) was utilized for imaging the cross-section of the oxides/oxyhydroxides layers. The surface profiles of the carbon steel samples were evaluated after immersion via confocal laser scanning microscopy (LEXT, OLS 3100) assisted by a table stable (T5-150).

The microscopy connected with drivers (OLYMPUS, MM6-ASPS) and (OLYMPUS, OLS 300).^58–60^ In addition, for an additional complete assessment of the surface after immersion, a scanning electron microscope (SEM) (Thermo – Phenom, Model: G5 XL) was operated on for morphological examination. In detail, this equipment was attached EDS for the compositional analysis of surface corrosion products with focus on (Fe, O, C, Na, N, and Cl chemical compounds). The results relating to these techniques that are not in the article are in the Supporting Information.

### 2.5 Rust Phase Analysis

After the electrochemical experiments executed by the authors,^21,34,35^ the carbon steel (ASTM A36) specimens used for rust phase analysis were carefully removed from the electrolyte and gently rinsed with deionized water to remove residual salts from the solution. This procedure was performed carefully to avoid disturbing or removing the corrosion products formed on the surface during exposure. The samples were subsequently dried under ambient laboratory conditions for approximately 0.5–1 min, with the temperature maintained between 200 and 250 °C at a distance of 15 cm, in order to prevent damage to the sample surface, It is worth noting that this surface preparation is carried out as quickly as possible to prevent the formation of oxides/oxyhydroxides on the material being evaluated, in this case, ASTM A36 carbon steel.. No mechanical cleaning, polishing, or chemical treatment was applied after the corrosion tests in order to preserve the integrity of the rust layer formed during the experiments. The corrosion products present on the steel surface were subsequently characterized directly by X-ray diffraction (XRD) and Raman spectroscopy to identify the crystalline phases of the oxides and oxyhydroxides formed under the tested conditions.

For the steel evaluation, the X-ray diffraction equipment (X’PERT PRO MPD PANALYTICAL) utilized to identify the phases in the carbon steel (A36), using this setup, data were collected from an angle of 40° to 110° (2Θ) degrees at a scan rate of 0.02 step-1 and scan size of 0.02. Cu–Kα beam with a current of 30 mA and a wavelength of 1.542 Å at a voltage of 40 kW was used in all tests, and the phases in the X-ray diffraction pattern obtained from the samples were identified by Xpert High Score software 3.0 and (PAN analytical, The Netherlands).^61^ Additionally, the XRD analyses determined the crystalline phases via Raman spectroscopy (inVia Qontor, Renishaw). Furthermore, two lasers with distinct wavelengths of the applied excitation line were applied in the infrared region (785 nm) and the visible region (532 nm). To assist this evaluation, an optical microscope with 100× objective was employed to determine the analysis zone.^62^ The microhardness was obtained by the Struers model Durmin and IVIUM Vertex hardness tester. The applied load time was 10 s and applied to the amount of 300 mN (HV 0.2). Ten hard points were taken from each sample, and their average was considered the microhardness.

To confirm the phases identified in the corrosion products, Mössbauer spectroscopy was employed. The spectra were collected in transmission mode at room temperature (≈298 K) using a SEE Co. Model W302 Resonant γ-ray Mössbauer Spectrometer. A radioactive ^57^Co source embedded in a rhodium matrix was used as the γ-ray source. The source was mounted on a velocity transducer operating in sinusoidal mode, with a velocity range from −12 to +12 mm s^–1^. The velocity scale was calibrated using a standard α-Fe foil, and the isomer shifts (δ) are reported relative to α-Fe at room temperature.

The spectra were fitted by the least-squares method using Lorentzian line shapes implemented in the NORMOS-90 software. For sample preparation, the specimens were mounted on an acrylic holder with a diameter of 0.5 cm, and a lead mask of the same diameter was used as a collimator for the γ-ray beam. Additional results obtained from this technique that are not discussed in the main text are provided in the Supporting Information.

## 3 Results and Discussion

### 3.1 Phases Analysis of Corrosion Product by X-ray Diffraction (XRD) Analysis

It is important to emphasize that the formation of the different corrosion products observed in this study cannot be attributed to variations in the electrolyte composition, since all experiments were performed in the same saline medium (3.5 wt % NaCl). Therefore, the differences detected in the relative intensity and distribution of oxide and oxyhydroxide phases are primarily associated with the presence of the protic ionic liquids and their interaction with the carbon steel surface.

In the blank condition, the corrosion process proceeds under unrestricted electrochemical dissolution of iron, typically resulting in the rapid formation of metastable phases such as lepidocrocite (γ-FeOOH), which are commonly reported as initial corrosion products in chloride-containing environments. However, when PILs are present in the solution, their adsorption on the metal surface can modify the local electrochemical environment by reducing the number of active dissolution sites and influencing the nucleation process of corrosion products. This adsorption effect may alter the balance between the formation of metastable and thermodynamically more stable phases, thereby promoting a different evolution pathway for the corrosion layer.

In particular, the presence of the inhibitors PIL 01 (2-HEAF) and PIL 02 (2-HDEAF) was associated with the detection of goethite (α-FeOOH), a phase widely recognized in the literature as a more compact and structurally stable corrosion product compared with lepidocrocite. The formation of goethite is often interpreted as a sign of maturation of the corrosion layer, in which less stable oxyhydroxides progressively transform into denser phases with lower permeability.

From a corrosion protection perspective, this transformation is highly relevant because the acicular and compact morphology typically associated with goethite can reduce the porosity of the corrosion layer and hinder the diffusion of aggressive species such as chloride ions toward the metal surface. Consequently, the presence of this phase may contribute to the development of a more protective interfacial barrier, limiting localized corrosion processes and slowing the overall corrosion kinetics. Therefore, the results suggest that the PILs evaluated in this study do not merely act as conventional adsorption inhibitors but may also influence the evolution and structural organization of the corrosion product layer. This combined effect, involving both surface adsorption and modification of oxide formation pathways, provides a plausible explanation for the improved inhibition efficiency previously reported for these compounds in electrochemical and gravimetric evaluations.

Figure 1 exhibits the X-ray diffraction (XRD) patterns of carbon steel samples oxidized after 24 h of immersion in a 3.5 wt % NaCl saline solution containing Protic Ionic Liquids (PILs) as corrosion inhibitors. In detail, the main corrosion products identified are lepidocrocite (γ-FeOOH) and magnetite (Fe_3_O_4_), accompanied by minor diffraction peaks corresponding to goethite (α-FeOOH). According to a more in-depth study on the formation of oxides and oxyhydroxides, it was observed that one phase was common to the results of the best inhibitors (PILs 01 and 02). Following this investigation and detailed study, it appears from the literature that the formation of goethite can act as a compact and dense physical barrier between layers of other oxides on carbon steel, in this case lepidocrocite and magnetite, details in the Discussion section and Supporting Information. Aiming to further elucidate the identification of corrosion products, this study examined whether variations in inhibitor concentration influence the evolution of the corrosion product layer on carbon steel. In this concern, XRD phase analysis, supported by SEM surface morphology and OM observations (Figures 1, 3, 4, and 5), shows that increasing the PIL concentration does not modify the qualitative phase composition, as the same three oxyhydroxide phases are detected at both 500 and 1000 ppm. While a reduction in the intensity of the lepidocrocite (γ-FeOOH) and magnetite (Fe_3_O_4_) diffraction peaks is observed at higher PIL concentrations, this effect cannot be attributed solely to a decrease in corrosion layer thickness. SEM and OM images reveal a concomitant reduction in surface roughness and a transition toward a more homogeneous and compact interfacial layer, indicating that the inhibitor alters the growth mechanism and spatial distribution of corrosion products rather than merely suppressing film thickness.

**Figure 1:**
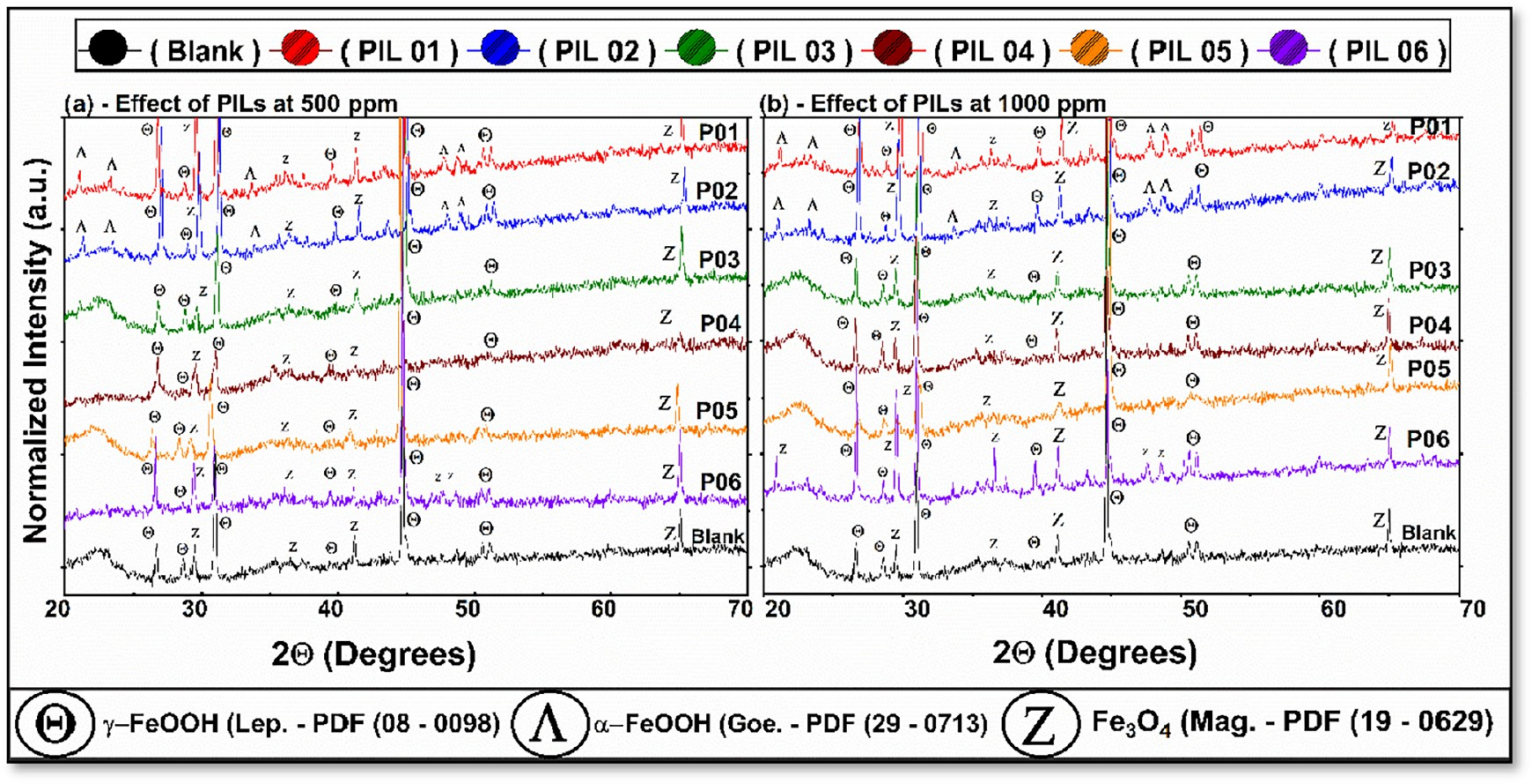
XRD patterns of the oxidized surface of ASTM A36 carbon steel after 24 h of exposure to 3.5 wt % NaCl solution containing different concentrations (500 and 1000 ppm) of each PIL.

This behavior is consistent with a concentration-dependent adsorption and passivation mechanism, in which increased availability of PIL species enhances surface coverage through interactions between the protonated cationic groups and the steel substrate, thereby restricting active dissolution sites and limiting oxide nucleation and growth. The combined structural and morphological evidence supports the conclusion that PIL concentration governs corrosion protection by modulating corrosion kinetics and surface passivation, rather than by simply producing thinner corrosion films.^63^ Nevertheless, complementary surface-sensitive and cross-sectional techniques, such as X-ray photoelectron spectroscopy (XPS) and cross-sectional FIB–SEM analysis, would provide further insight into the chemical composition and thickness distribution of the protective layers. This study evaluates the approach based solely on the FIB/SEM technique, demonstrating the significance of corrosion product layer formation.

In the initial region of the spectral pattern, the lepidocrocite peak (≈20°) is barely discernible at inhibitor concentrations between 500 and 1000 ppm. Subsequently, two peaks characteristic of goethite, located at approximately 23° and 25°, are observed exclusively for PILs 01 and 02 and are absent in the blank sample and in the other corrosion inhibitors evaluated in this thesis. This behavior is accompanied by an increase in the intensity of the initial peaks associated with the goethite phase. Such a response may be attributed to the optimized inhibitor concentration, which promotes the preferential formation of specific iron oxide phases on the metal surface, likely as a result of the blocking effect induced by the corrosion inhibitors.^64,65^

At the angle of 30°, an increase in the peaks associated with lepidocrocite and magnetite is observed. At close to 33, characteristic peaks of goethite are present; however, upon reaching the maximum concentration, a reduction from two peaks to a single one is noted, a pattern also observed in the inhibitors (PIL 01/02). At 35, a minor magnetite phase is present in all samples, although it is not clearly distinguishable at either concentration. Certain phases may exhibit limited variation in response to changes in concentration, for example varying from 500 to 1000 ppm, while others show significant changes due to the nature of oxide or oxyhydroxide formation.

This phenomenon can be attributed to the amount of inhibitor that is responsible for the formation of a protective layer on the material and that over time this layer evolved into the oxides/oxyhydroxides formed depending on the environment. At 40, the presence of lepidocrocite and magnetite phases can be observed. As the concentration increases, the magnetite phase tends to become more prominent, likely due to its mode of formation, which is more external relative to the complex oxide layer formed on the metallic surface following corrosion (see details in Table 2).^37,66^ At 45, the presence of the lepidocrocite phase is distinctly evident, confirming its role as one of the predominant phases forming on the material’s surface under the given conditions.

**Table 2:** X-ray Diffraction Data for Iron Oxides and Oxyhydroxides in the Literature

Compounds	Angle (2Θ) (Thesis)	Angle (2Θ) (Literature)	References
Magnetite	(29), (36), (42), (62).	30,35,43,45, 57,62,72,75	^67^
		30,32,45,60	^68^
		30,32,36, 42,54,56,62,72	^69^
		30,36,57,63	^70^
		30,36,42,56, 57,62	^71^
		30,35,45,55,62	^72^
		30,42,62,72	^36^
		31,46,57,64	^73^
Goethite	(21), (25), (33), (48), (50)	35,36,37,41,45,55,61,62,68,70,71	^67^
		20,25,31,38,40,42,46,48,55,58	^74^
		21,26,33,58,62	^75^
		21,25,33,38,40,43,47,48,50	^68^
		21,31,37,50	^71^
		21,22,33,54,55,58	^72^
		21,35,37,48,50	^36^
		21,33,37,58,60	^73^
Lepidocrocite	(19), (25), (27), (31), (38), (45), (51), (60)	27,29,36,37,39,43,47,49,50,60,68	^76^
		15,22,38,40	^75^
		15,32,36,47,53	^70^
		31,34,44,53,54,60	^77^
		38,42,44,49	^36^
		15,24,38,40,47,53,60,68,80	^73^

This phase is typically associated with intermediate stages of corrosion in iron-based alloys and is often used as an indicator of oxidation progress. As concentration increases, a marked enhancement in the intensity of the lepidocrocite signal is observed, suggesting a direct relationship between concentration and phase development. This observation supports the hypothesis that optimized concentrations can effectively promote the formation of specific oxide and oxyhydroxide species on the surface of the material. The increased intensity within the same exposure period implies a more efficient oxidation process, potentially driven by the availability of reactive ions or by altered electrochemical dynamics.

This result reinforces the importance of concentration as a key parameter in controlling surface transformation processes and highlights the significance of lepidocrocite as a diagnostic phase for evaluating the efficiency of oxide formation under various treatment conditions (details in Table 2). In relation to the phases identified at 47 and 48, the presence of goethite oxyhydroxide is confirmed, which is another common and thermodynamically stable iron corrosion product. Notably, as concentration increases, a progressive enhancement of the second peak corresponding to goethite is detected, indicating not only the formation but also the growth or densification of this phase.

This behavior suggests that higher concentrations facilitate conditions favorable to goethite precipitation, potentially through increased local alkalinity or altered redox equilibria at the surface. At 51, the lepidocrocite phase reappears with greater intensity, and this increase is accompanied by the emergence of multiplet features in the spectrum.These multiplets are indicative of structural disorder or the coexistence of multiple oxidation states within the oxide layer, which may arise from overlapping growth regimes or mixed-phase boundaries.

The observation of such spectral complexity highlights that the oxide layer undergoes structural modifications as a function of concentration, further supporting the premise that optimized concentrations play a critical role in modulating the surface chemistry and the structural organization of corrosion products. At 60, the lepidocrocite phase remains consistent across both concentrations (500 to 1000), indicating a saturation point beyond which further increases in concentration do not significantly influence its formation. At 65, magnetite intensity remains nearly unchanged between concentrations, suggesting that its formation is less responsive to concentration variations. These observations underscore that while certain phases are notably affected by concentration changes, others exhibit relative stability.

Table 2 presents a detailed summary illustrating the correspondence between the identified phases and those reported in the literature concerning the identification of oxides and oxyhydroxides. The primary objective was to determine the predominant phases of the three main oxides/oxyhydroxides, Magnetite, Goethite, and Lepidocrocite, formed on the surface, both in the absence and presence of inhibitors (PILs).

### 3.2 Macroscopic Morphology and Phase Analysis of Rust

Before addressing the specific iron oxide and oxyhydroxide phases identified on the carbon steel surface, the optical micrographs obtained after 24 h of immersion in a 3.5 wt % saline solution were first examined (additional details are provided in the Supporting Information). This preliminary visual assessment is a critical step in corrosion studies, as it allows the identification of surface heterogeneities, such as localized corrosion products, discoloration, or deposits, thereby enabling the accurate selection of representative regions for subsequent Raman spectroscopy analysis (Figure 2). By guiding the positioning of the laser beam toward well-defined areas, this approach ensures that the acquired spectra are directly associated with specific corrosion features rather than average surface responses.

**Figure 2:**
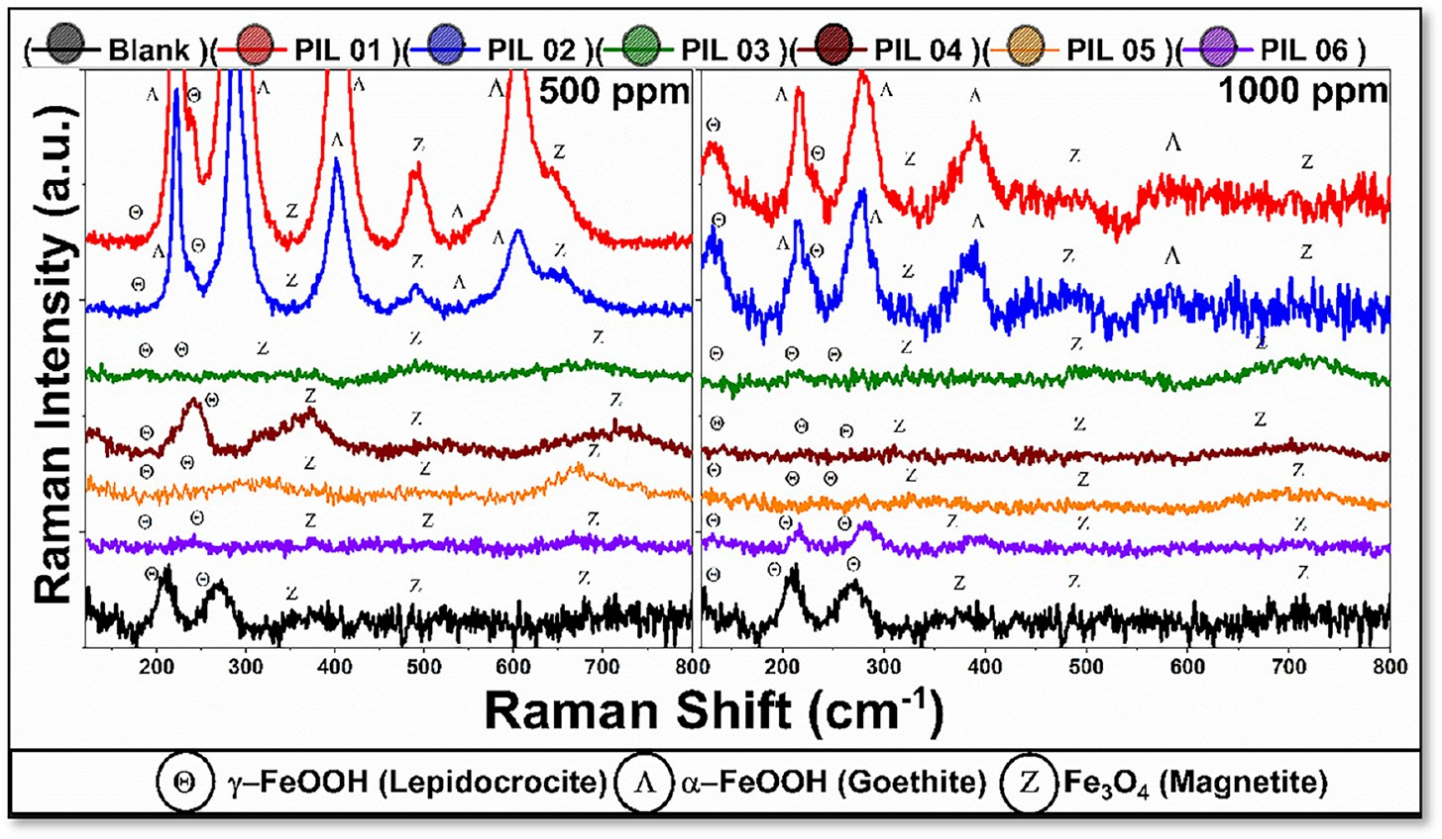
Raman spectra of the ASTM A36 steel surface after 24 h of exposure in 3.5 wt % NaCl solution containing all tested protic ionic liquids (PILs).

The primary objective of this analysis was to evaluate the macroscopic appearance and surface coverage of the carbon steel surface after 24 h of exposure and, based on these observations, to define representative regions for Raman spectral acquisition. Table 3 summarizes the Raman spectral features obtained in this study from carbon steel samples exposed for 24 h to a 3.5 wt % NaCl solution containing the different corrosion inhibitors investigated, each evaluated at two concentrations (500 and 1000 ppm). This comparison was specifically designed to assess whether variations in inhibitor concentration under the present experimental conditions influence not only the presence but also the relative distribution of iron oxide and oxyhydroxide phases formed during the early stages of corrosion.

**Table 3:** Raman Wavenumbers of Iron Oxides and Oxyhydroxides as Reported in the Literature^a^

Compounds	WN*(cm^–1^) (This work)	WN*(cm^–1^) (Literature)	ref
Magnetite	(320–370)	670,500	^78^
		249,320,376	^79^
		550,674	^80^
	(480–540)	350,535,672	^81^
		230	^46^
	(650–720)	320,540,670	^82^
		210,300,400,670,700	^28^
		300,500,670,700	^83^
Goethite	(200–230)	200,230,250,300,390,500,550,700	^78^
		428,495,505.	^79^
	(260–300)	-	^80^
	(390–400)	220,250,300,400,500,550,650	^81^
	(550)	600,610	^46^
	(580–600)	244,299,385,480,548,681	^82^
		240,300,380,400,480,550	^83^
Lepidocrocite	(170–180)	250, 300, 400, 500, 550	^78^
		570	^79^
	(200–220)	180,200,400,528,646	^80^
		150,245,250,290,300,412	^82^
	(260–280)	220,250,310,345,380,525	^83^

a WN for Wavenumber.Source.

At the higher inhibitor concentration (1000 ppm), the Raman spectra exhibit more clearly defined and distinguishable features, with noticeable changes in the characteristic bands associated with lepidocrocite (γ-FeOOH), magnetite (Fe_3_O_4_), and goethite (α-FeOOH). These spectral differences suggest that the inhibitor concentration plays a significant role in modulating the corrosion pathways and, consequently, the nature of the corrosion products formed on the steel surface.

To support these semiquantitative observations, peak normalization and intensity ratio analysis were employed, allowing for a more reliable comparison among spectra acquired under different experimental conditions and minimizing the influence of local signal fluctuations.

For PILs 01 and 02, a relative increase in the normalized intensity of lepidocrocite-related bands, particularly within the 130–150 cm^–1^ spectral region, was observed as the inhibitor concentration increased from 500 to 1000 ppm. This trend indicates a concentration-dependent modification of the corrosion product layer, suggesting that higher inhibitor concentrations may promote the preferential stabilization or accumulation of lepidocrocite during the initial corrosion stages. Such behavior may be associated with the adsorption of PIL species on the steel surface, which can locally alter ion transport and redox conditions, thereby influencing the nucleation and growth of specific oxide/oxyhydroxide phases.

Regarding the phases identified in the Raman spectra, a reduction in the intensity of bands attributed to goethite was observed at approximately 210, 260, 380, 400, and 600 cm^–1^ (Figure 2). This behavior may be associated with the relatively slow formation kinetics of goethite.

Previous studies report that this phase typically forms after longer exposure times, often exceeding 170 h (≈7 days), through the gradual transformation of precursor iron oxide and oxyhydroxide phases. Under shorter immersion times, the evolution of corrosion products may be limited, which can reduce the detectability of well-defined Raman bands related to goethite. A similar trend was observed for magnetite, with a decrease in band intensity near 500 and 700 cm^–1^ (Figure 2). This behavior suggests a lower extent of oxide formation on the carbon steel surface in the presence of the inhibitors. As the inhibitor concentration increases, the adsorption of protic ionic liquid (PIL) molecules can contribute to the formation of a protective surface film, which may limit the growth of corrosion products and lead to a more compact surface layer.^37^

The higher inhibitor concentration may partially contribute to the development of a more compact and protective interfacial layer on the steel surface; however, this interpretation cannot be established from corrosion product characterization alone. Instead, it is supported in a qualitative manner by electrochemical trends previously reported by the authors for similar protic ionic liquid systems, where increased inhibitor concentrations were associated with open circuit potential stabilization and higher impedance responses. These electrochemical features are commonly interpreted as indicative of a reduced metal–electrolyte interaction and improved surface coverage. Within the scope of the present study, which is primarily focused on corrosion product evaluation, such electrochemical behavior provides a contextual framework to interpret the observed evolution of oxide and oxyhydroxide phases. In this sense, the relative persistence of lepidocrocite-related features may be associated with changes in the interfacial conditions during oxidative reactions in the saline environment, rather than being considered direct evidence of enhanced film compactness.

The enhanced concentration of inhibitors may, therefore, create a favorable environment for the development of protective coatings, which can play a significant role in reducing corrosion progression under such conditions. However, according to the literature, the presence of certain oxide phases on the metal surface tends to promote the permeation of chloride ions (Cl^–^) into the outermost layer of carbon steel. This can result in more severe degradation, particularly when these oxides are not compact enough to provide effective protection at the base of the oxide layer.^38^

Additionally, Table 3 presents a detailed summary of the correspondence between the identified phases and those reported in the literature regarding the characterization of oxides/oxyhydroxides. The primary objective was to identify the predominant phases of the three main oxides/oxyhydroxides magnetite, goethite, and lepidocrocite, formed on the surface of carbon steel (A36) after 24 h of immersion, both in the absence and presence of inhibitors (PILs) at concentrations of 500 and 1000 ppm. It is important to note that the obtained values are consistent with those reported in the literature and demonstrate variations according to concentration.

This was the primary objective of the present evaluation, as a significant variation associated with a higher inhibitor concentration would have direct implications for the final production outcome (as illustrated in Figure 2). Based on the data presented in Table, it is evident that when compared to literature the values obtained from the spectral peaks correspond to oxides formed on the metal surface. For magnetite, phase values were observed with wavelengths between these ranges in this work (320–370 cm^–1^), (480–500 cm^–1^), (650–720 cm^–1^). In the literature, there is (320 cm^–1^),^79^ (350 cm^–1^),^81^ (320 cm^–1^)^82^ for the values around (300 cm^–1^). Also, in the literature (500 cm^–1^),^78^ (550 cm^–1^),^80^ (535 cm^–1^),^81^ (540 cm^–1^),^82^ (500 cm^–1^)^83^ for the values around (480 to 540 cm^–1^).

Continuing, about the data found in the literature, the wavelengths, for the values around (670 cm^–1^),^78^ (674 cm^–1^),^80^ (672 cm^–1^),^81^ (670 cm^–1^),^82^ (670–700 cm^–1^),^28^ (670–700 cm^–1^)^83^ for values around (650 to 720 cm^–1^). For goethite, phase values were observed with wavelengths between these ranges in this work (200–230 cm^–1^), (260–300 cm^–1^), (390–400 cm^–1^), (550 cm^–1^) and (580–600 cm^–1^). In the literature, there is (200 and 230 cm^–1^),^78^ (220 and 250 cm^–1^),^81^ (244 cm^–1^)^82^ and (240 cm^–1^)^83^ for the values around (200–230 cm^–1^). Also, in the literature (250 and 300 cm^–1^),^78^ (250 and 300 cm^–1^)^81^ and (300 cm^–1^)^83^ for the values around (260–300 cm^–1^). Remaining, about the data found in the literature, the wavelengths, for the values around (390 cm^–1^),^78^ (428 cm^–1^),^79^ (400 cm^–1^),^81^ (385 cm^–1^)^82^, and (380 and 400 cm^–1^)^83^ for values around (390–400 cm^–1^). Remaining, about the data found in the literature, the wavelengths, for the values around (550 cm^–1^),^78^ (550 cm^–1^),^81^ (548 cm^–1^)^82^ and (550 cm^–1^)^83^ for values near (550 cm^–1^). Remaining, about the data found in the literature, the wavelengths, for the values around (650 cm^–1^),^81^ (600 cm^–1^),^46^ (681 cm^–1^),^82^ and (550 cm^–1^)^83^ for values near (580–600 cm^–1^).

For lepidocrocite, phase values were observed with wavelengths between these ranges in this work (170–180 cm^–1^), (200–220 cm^–1^), (260–280 cm^–1^). In the literature, there is (180 cm^–1^)^80^ and (150 cm^–1^)^82^ for the values around (170–180 cm^–1^).

Also, in the literature (200 cm^–1^),^80^ (245 cm^–1^),^82^ (220 cm^–1^)^83^ for the values around (200 and 220 cm^–1^). Continuing, about the data found in the literature, the wavelengths, for the values around (250 cm^–1^),^78^ (245, 250, 290, and 300 cm^–1^),^82^ (670–700 cm^–1^),^28^ (250–310 cm^–1^)^83^ for values around (260–280 cm^–1^). It should be noted that several Raman bands attributed to lepidocrocite partially overlap with characteristic vibrational modes of other iron oxides and oxyhydroxides, particularly goethite and magnetite, within similar spectral regions. In particular, the bands observed in the ranges of approximately 170–180 cm^–1^ and 250–280 cm^–1^ have been reported to coincide with modes associated with goethite, while contributions from magnetite may also be present in the low-wavenumber region below 300 cm^–1^.

As a result, the assignment of these bands to lepidocrocite should be interpreted with caution, especially when phase coexistence is expected during corrosion. In this context, phase identification was based on the combined evaluation of band positions, relative intensities, and their evolution with inhibitor concentration and exposure time, rather than on isolated spectral features. This multicriteria approach reduces the uncertainty associated with band overlaps and supports a more reliable interpretation of the corrosion product composition.

Therefore, given the results described and discussed above, it can be satisfactorily concluded that the phases identified in the immersion tests using the Raman technique are indeed associated with the formation of oxide and oxyhydroxide compound specifically Magnetite, Goethite, and Lepidocrocite. This conclusion is strongly supported by the correspondence between the Raman shifts observed in this study and those consistently reported in the literature across various wavenumber ranges. The identification of these compounds, based on their characteristic vibrational modes, confirms the reliability of the experimental procedure and reinforces the accuracy of the spectral interpretation. The consistent match with previously documented data validates the presence of these phases and highlights their relevance in the corrosion processes under the tested conditions (details in Table 3).

Magnetite (Fe_3_O_4_) is a widely observed phase in the transformation of corrosion products that form on carbon steel, particularly when the material is exposed to saline environments. Its gradual accumulation over time can be attributed to multiple underlying factors related to the thermodynamic and chemical structure of the system. Magnetite is characterized by its low solubility in aqueous media and its superior thermodynamic stability compared to other commonly formed phases, such as lepidocrocite (γ-FeOOH) and goethite (α-FeOOH) details in Figure 2. These properties enable magnetite to persist above the material surface, such as steel, and become predominant as the corrosion process progresses. In environments with elevated chloride concentrations, such as the 3.5 wt % solution utilized in this thesis, the initial corrosion products, particularly lepidocrocite, may gradually transform. These modifications/transformations may result from reduction processes and internal structural reorganization, ultimately leading to the formation of more stable phases such as magnetite.

Consequently, magnetite is frequently observed as a later-stage product in the corrosion process, reflecting the system’s evolution under sustained exposure to aggressive conditions.^37^ Furthermore, in systems containing corrosion inhibitors such as PILs, the selectivity of oxide formation may be influenced by chemical interactions between the inhibitors and the metal surface.

In the case of PIL 03, the promotion of magnetite formation may be associated with its ability to alter the electrochemical equilibrium at the metal/solution interface, thereby stabilizing this intermediate phase. Another important factor is the local reduction of the redox potential, which may favor the conversion of less stable oxidic phases into magnetite, delaying the formation of more hydrated or less protective layers, such as ferrihydrite and lepidocrocite.

Therefore, the increasing presence of magnetite over time may be an indication of the dynamics of transformation of corrosion products in a saline medium, reinforced by the action of inhibitors in modulating the stability of the phases formed on the surface of carbon steel.

In addition, for the inhibitor PIL 04, a considerable reduction in the lepidocrocite (250 cm^–1^) and magnetite (350–400 cm^–1^) phases was observed in the Raman experiments. Overall, this result may be attributed to the influence of the inhibitor presence on the formation and stability of corrosion products on the carbon steel surface. In fact, as a metastable phase in saline environments, lepidocrocite may undergo transformations over time, particularly in the presence of compounds that alter the electrochemical potential at the metal/solution interface.

The simultaneous reduction of magnetite suggests that PIL 04 interferes with the oxidative process, inhibiting both the initial formation of this phase and its conversion from less stable intermediates. In addition, the inhibitor PIL 05 also showed a reduction in the magnetite phase, with a wavelength close to 700 cm^–1^, reinforcing the hypothesis that the addition of inhibitor compounds significantly alters the kinetics of formation and transformation of oxides on the surface, as shown in Figure 2.

The reduction in magnetite intensity may indicate that PIL 05 limits the progression of the oxidation process, hindering the nucleation and growth of this phase. This effect may favor the formation of other corrosion products or less crystalline oxide phases on the steel surface. Additionally, it reflects the predominance of specific phases in saline media. Notably, unlike the other protic ionic liquids, PIL 06, characterized by the longest carbon chain evaluated, a considerable increase in the formation of lepidocrocite (210–280) and magnetite (380–400). Overall, this result may be directly associated with the distinguishing feature of this compound: the length of the carbon chain in the anionic fraction of the protic ionic liquid used in a 3.5 wt % NaCl solution.

### 3.3 Evaluation of Surface Topography and Microscopic Morphology of Rust Layers: Surface Features and Cross-Sectional Elemental Analysis

The morphological and dimensional diversity observed after 24 h of exposure to a 3.5 wt % NaCl solution was analyzed using Scanning Electron Microscopy (SEM) and Energy Dispersive Spectroscopy (EDS), as illustrated in Figures 3, 4, and 5. In the blank sample, used as a control system without the addition of corrosion inhibitors, the carbon steel surface (ASTM A36) exhibited a heterogeneous distribution of corrosion products characterized by distinct morphologies.

**Figure 3:**
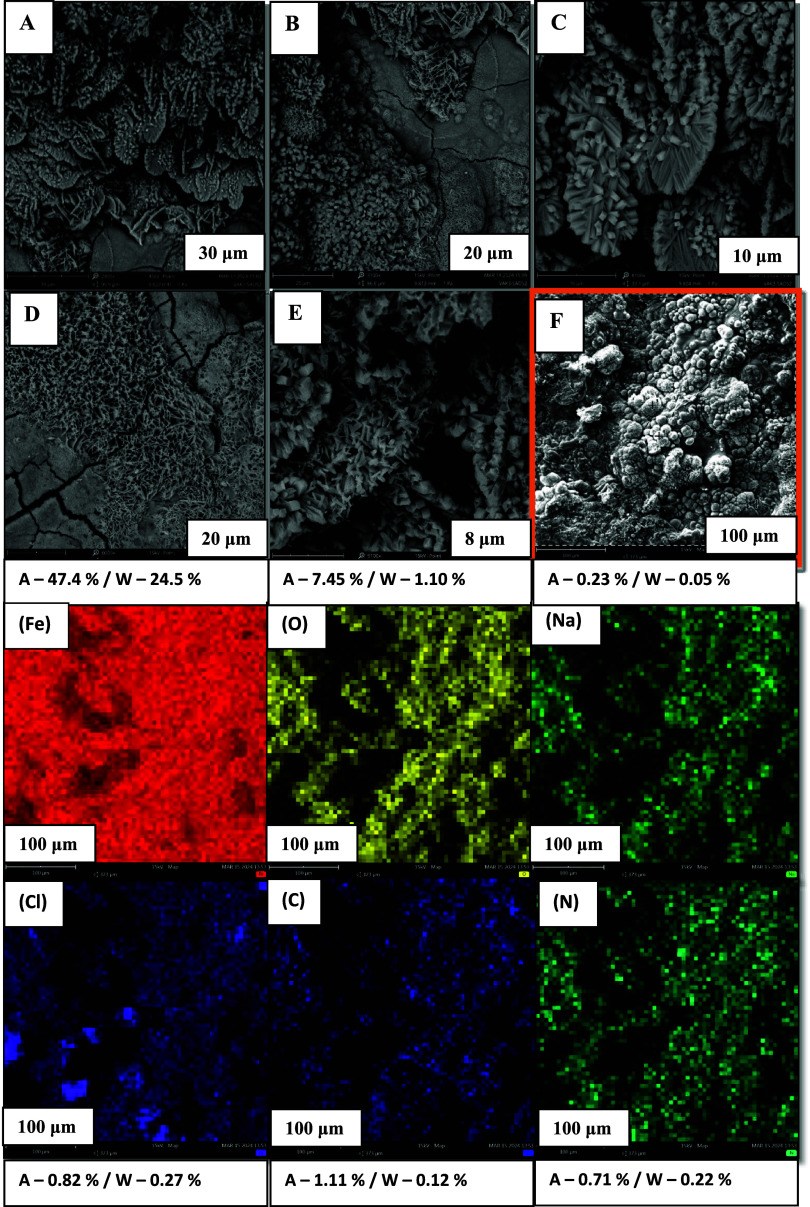
SEM images of regions (A–F) and Energy Dispersive Spectroscopy (EDS) (Sec. F) on the carbon steel surface after 24 h immersion without corrosion inhibitors.

**Figure 4:**
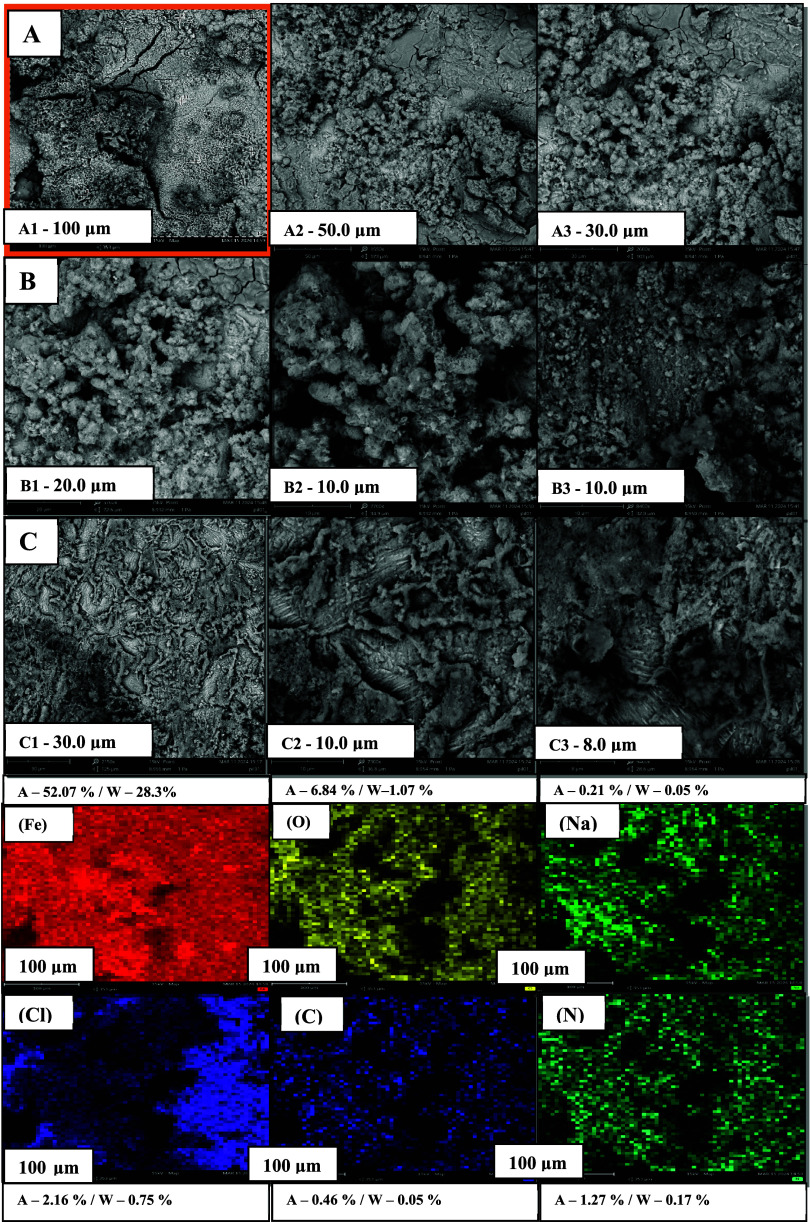
SEM images of regions (A–C) and Energy Dispersive Spectroscopy (EDS) (Sec. A) on the carbon steel surface after 24 h immersion with PIL01 (HEAF).

**Figure 5:**
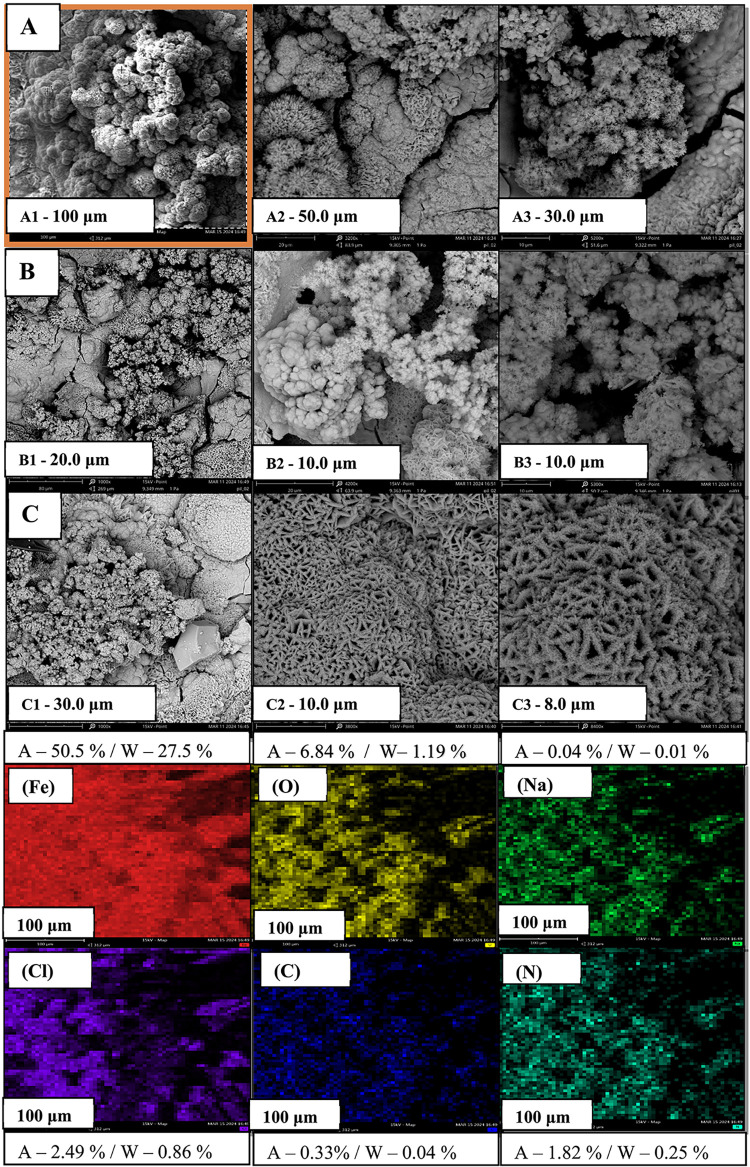
SEM images of regions (A–C) and Energy Dispersive Spectroscopy (EDS) (Sec. A) on the carbon steel surface after 24 h immersion PIL02 (HDEAF).

These products correspond to specific iron oxide and oxyhydroxide phases, namely lepidocrocite (γ-FeOOH) and magnetite (Fe_3_O_4_), both presenting unique morphological features and particle sizes (Figures 3, 4, and 5). A key observation arising from this analysis concerns the high porosity of the corrosion layer, which promotes the formation of microcracks and localized structural degradation, indicating the presence of a mechanically fragile and poorly adherent protective film.

In contrast, the samples treated with Protic Ionic Liquids (PILs) developed denser and more compact oxide layers, predominantly composed of goethite (α-FeOOH). The presence of this phase and the observed morphology may indicate the formation of a more uniform corrosion product layer on the steel surface; however, it is important to note that electrochemical measurements or weight-loss tests were not performed in this section to directly confirm the improvement in corrosion resistance. The morphological characterization followed the criteria and background summarized in Table 4, compiled from key references that discuss corrosion products with a specific focus on iron oxides and oxyhydroxides.^54,84^

**Table 4:** Corrosion Produces Information about Morphology Based on Literature

		Morphology			
Iron oxide/oxyhydroxide	Colors	^89^	^85^	^90^	^45^
Goe—(α FeOOH)	Yellowish to reddish to dark	Acicular	Star, hexagons,bipyramids, cubes.	Cloud, thin, flat sheet, cotton-balls	Needle, shaped, laths whiskers.
Lep—(γ-FeOOH)	Black to Dark Gray	Laths	Tablets, plates, diamonds, cubes	Thick plates, sandy, thick	Sandy grain, worm nest
Mag—(Fe_3_O_4_)	Strong red to red to orange	Octahedra	Octahedral, rhombic dodecahedra.	Flat and dark layer, circular	Blackish circular rings

In sum, specifically, the SEM image of the blank/control sample reveals a significant accumulation of this material on the carbon steel surface indicating the aggressiveness of the saline environment against carbon steel.

From a corrosion resistance perspective, the differences in morphology and phase composition are particularly relevant. Lepidocrocite (γ-FeOOH), commonly observed in the blank system, typically forms porous and poorly adherent layers that facilitate electrolyte penetration and sustain the corrosion process. In contrast, the formation of goethite (α-FeOOH) in the presence of PILs is generally associated with more compact and structurally stable corrosion products, which can act as a more effective barrier to ionic transport at the metal and solution interface.

Furthermore, variations in PIL concentration may influence the nucleation and growth of these oxide phases, thereby affecting the morphology and compactness of the corrosion layer. At higher inhibitor concentrations, the increased availability of adsorbed ionic species at the interface may promote the development of more uniform and adherent oxide films, contributing to improved surface coverage and enhanced resistance to localized corrosion processes.

These observations highlight the important role of PIL-mediated interfacial interactions in modulating corrosion product formation and, consequently, the overall protective behavior of the oxide layer formed on carbon steel.

In Figure 3 (sections A–C), a dense cluster of magnetite crystals resembling sand-like particles can be seen, forming a layered arrangement. The upper portion of the image displays the typical “worm nest” structure of lepidocrocite,^85^ while the central region shows coral-like formations of magnetite, representing intercalated growth of oxide phases formed during corrosion.

In section C of the micrograph, a greater presence of magnetite crystals (resembling sand-like structures) is observed. Immediately beneath these, a cluster resembling a bird’s nest can be seen forming a conglomerate, accompanied by sharp flakes adjacent to a significant surface crack. According to the literature, such oxide and oxyhydroxide formations typically exhibit high permeability when formed on metallic surfaces, particularly with respect to chloride ion ingress.

This elevated permeability may be associated with the emergence of surface cracks and the relatively loose packing of these crystalline structures. Furthermore, in the vicinity of the crack, a substantial accumulation of worm-like formations characteristic of lepidocrocite is also evident. Still concerning sections D–F, the micrographs reveal distinct morphological characteristics of the corrosion products formed on carbon steel after exposure to the saline medium.

Specifically, sections D and E display acicular and lamellar morphologies, typically associated with lepidocrocite (γ-FeOOH) and magnetite (Fe_3_O_4_), indicating the formation of a porous and heterogeneous corrosion layer susceptible to crack propagation and reduced protective effectiveness, regardless of this observation a more comprehensive investigation is needed to understand how chloride would be influenced by accessing the surface in the presence of these oxides.

Section F provides a broader view of the oxide layer on the surface, characterized by a denser structure resulting from the accumulation and coalescence of previously identified phases. This morphology suggests moderate adhesion combined with significant porosity, favoring chloride ion penetration and promoting progressive surface degradation over time.

In Figure 3, an accumulation similar to that observed in Section A can be identified. However, this micrograph reveals a comparatively “cleaner” region of the sample, characterized by a reduced presence of conglomerates. Within this area, it is evident that the electrolyte induces accumulation on the surface, accompanied by porous and visible cracks. The formation of magnetite is observed, exhibiting granular and geometric crystalline structures.

The lepidocrocite phase appears in diverse morphologies, including bird’s nest and worm’s nest patterns, as well as plate-like, needle-like, and globular formations. Sections C provide more detailed views: Section C focuses on the crystalline encasements associated with magnetite, while this section highlights the bird’s nest morphology and needle-like structures characteristic of lepidocrocite, which facilitate significant permeation of these oxides/oxyhydroxide.

Remaining the discussion of Figure 3, the elemental composition analysis conducted through Energy Dispersive Spectroscopy (EDS), presented in the lower region of the figure, confirmed the surface homogeneity following immersion while revealing localized areas with elevated concentrations of specific compounds on the carbon steel surface. Overall, the EDS elemental mapping depicts the spatial distribution of O, Fe, Na, N, C, and Cl, providing meaningful insights into the relationship between the formation of distinct corrosion products (oxyhydroxide phases) and the preferential accumulation of these elements across the corroded surface.

According to the results obtained, the oxygen content increased to 7.5%, while the concentrations of Fe, Cl, and N decreased to 47.2, 0.82, and 0.71%, respectively, after the exposure period in the saline medium without corrosion inhibitors. At first, the observed increase in oxygen is indicative of the early formation stages of lepidocrocite (γ-FeOOH) and magnetite (Fe_3_O_4_), which correlates with the reduction of surface iron content to values below 50%.^27,46,86,87^ The coexistence of this oxide and oxyhydroxide phases in the absence of PILs suggests their active role in the selective adsorption of chloride species, thereby influencing the structural and transport properties of the corrosion product layer, particularly regarding permeability and substrate degradation.

In the blank/control sample, localized chloride enrichment was detected in specific regions predominantly associated with lepidocrocite and magnetite, reinforcing their involvement in the initiation and propagation of localized corrosion phenomena. Figure 3 illustrates six main elemental maps in which iron, highlighted in red, dominates the surface evaluated due to its role in corrosion product formation, as the main component in the oxides/oxyhydroxides composition.

Nonetheless, regions of lower Fe concentration coincide with areas of reduced oxygen, nitrogen, sodium, carbon, and chlorine, likely corresponding to oxide phases with distinct elemental affinities. The correlation between chloride enrichment and the formation of lepidocrocite and magnetite is influenced by the proportion of [Cl]/[Fe], where higher ratios favor lepidocrocite formation and decrease magnetite intensity. Chloride presence, therefore, critically affects iron oxidation and the stability of corrosion products.^88^

Lastly, sodium, represented in green, appears broadly but diffusely distributed across the analyzed surface, indicating its weaker electrostatic interaction with the iron substrate compared to chloride ions. This diffuse distribution suggests that sodium remains more mobile within the corrosion layer, without forming stable compounds or strong adsorption sites on the metallic surface. In contrast, nitrogen and carbon exhibit lower concentrations, occupying larger unreacted regions, which may correspond to areas where corrosion reactions are less advanced or where organic residues are present over the surface.

The morphological and compositional analyses indicate that the blank sample develops a porous, heterogeneous and amorphous CP layer, mainly composed of lepidocrocite and magnetite. These phases, while characteristic of carbon steel corrosion in saline media, are associated with variable protective capacities. The localized chloride enrichment observed further contributes to the destabilization of these oxides, promoting the early onset and propagation of corrosion processes on the carbon steel surface.^27,46,86,87^

Based on the provided Table 4, the morphology of various iron oxide/oxyhydroxide corrosion products is highly diverse, ranging from acicular (α-FeOOH, Goethite) and laths (γ-FeOOH, Lepidocrocite) to octahedra (Fe_3_O_4_, Magnetite), with supplementary descriptions including star, needle, thick plates, and worm nest shapes. This variation is further correlated with distinct color characteristics, such as the yellowish to dark red of Goethite and the strong red to orange of Magnetite.

The identification of these specific corrosion products and their characteristic morphologies is critically important within the study of corrosion, as the physical structure of the rust layer directly influences the protective properties, stability, and growth mechanisms of the film.

Furthermore, accurate morphological analysis, often achieved through microscopic techniques (as referenced in the section titles), provides key insights into the environmental conditions (e.g., humidity, pH, and presence of specific anions) that dictated the corrosion process, thus enabling a mechanistic understanding of the material degradation and guiding the development of effective corrosion mitigation strategies.

To enable a more accurate evaluation of the oxides identified through scanning electron microscopy (SEM), each magnification level was designated as A1 (80 μm), A2 (20 μm), and A3 (10 μm) for the respective regions A, B, and C. Figures 4 and 5 illustrate the carbon steel surface after exposure to the inhibitors PIL 01 and PIL 02. The primary oxide phase identified was goethite (α-FeOOH), typically observed in cotton ball-like, grassy, or needle-like morphologies.^37,38,64^

Based on the oxides detected in Figure 4, Section A displays the initial development of goethite, primarily in the upper-left and central regions, exhibiting a diffuse, cloud-like morphology indicative of early stage formation frequently associated with lepidocrocite platelets arranged in “bird nest” configurations.^37,38,64^

Furthermore, upon initial evaluation of the micrographs, a significant accumulation of a specific formation on the material is observed: goethite in the center of the micrograph (divided into sections A1–A3). This accumulation forms conglomerates with a morphology identified in the literature as “cotton balls” due to the evolution of corrosion products on the surface of the carbon steel. In detail, in those sections, these structures generate a massive/dense structure capable of offering partial protection to the underlying surface over time, restricting possibly the permeation of ions into the steel matrix, mainly chloride and sulfate.

Acutely, in section A1, extensive cracking was observed near the lepidocrocite and magnetite phases; this occurrence may be associated with the type of structure that these oxides form on the material, considering that they are considerably more porous than the formation of goethite, for example. In detail, lepidocrocite presented morphologies similar to “worm nests” and “bird nests,” while magnetite occurred as geometric crystals, similar to sand, in the lower regions.

Section A2 revealed overlapping goethite structures resembling “cotton balls,” while Section A3 displayed crystalline magnetite associated with regions of goethite and lepidocrocite exhibiting “worm nest” morphologies.^45,85^ In Figure 4, particularly in Section B1, lepidocrocite (γ-FeOOH) appeared with its characteristic cited morphology typical of saline environments with elevated NaCl levels. The region showed marked porosity, fissures, and stomates, evidencing structural heterogeneity relevant to corrosion assessment. Section B2 confirmed the coexistence of goethite (α-FeOOH), lepidocrocite, and magnetite (Fe_3_O_4_), the latter forming sandy-like crystals within the same surface. Voids and cracks between goethite and magnetite conglomerates acted as diffusion pathways for chloride, hydroxyl, and carbonate ions during prolonged exposure.^91–93^ Section B3 further detailed distinct oxide morphologies under higher magnification.

In Section C, a barrier-like edge composed of oxides and oxyhydroxides was detected, likely resulting from accumulated laminar lepidocrocite platelets, goethite “cotton balls,” and magnetite crystals. Sections C2 and C3 showed compact oxide conglomerates forming a dense, protective layer limiting chloride penetration. Compared with the blank sample, PILs 01/02 produced surfaces with minimal cracks, supporting the inhibitors’ role in stabilizing the corrosion-product layer. For PIL 01, elemental data after 24 h revealed 52% Fe and 2.16% Cl, attributed to extended exposure enhancing chloride adhesion and iron dissolution. Despite high chloride content, no localized accumulations were observed, indicating homogeneous distribution associated with lepidocrocite formation. Oxygen (6.84%) and carbon (0.46%) decreased, while nitrogen rose to 1.27%, suggesting inhibitor adsorption via amine groups.^21,35^

The elemental distribution demonstrated that iron predominated, while oxygen, nitrogen, and sodium displayed broadly diffused patterns with localized deficiencies due to oxide heterogeneity. Chlorine, represented in purple, was uniformly dispersed, entrapped within denser oxide layers such as goethite, which can act as diffusion barriers. Nitrogen appeared evenly distributed, associated with the adsorbed inhibitor species, contrasting the blank sample’s deficiency. Carbon presented minimal contribution. The principal iron oxyhydroxide correlated with chloride accumulation was lepidocrocite (γ-FeOOH), a metastable phase prone to transformation into maghemite under chloride influence, thus threatening long-term structural integrity by promoting continuous substrate deterioration.^88^

Considering that the primary objective of this study is to identify the oxides present on the carbon steel surface, the results obtained after 24 h of immersion in a saline solution containing the second most effective corrosion inhibitor (PIL 02) reveal significant morphological and compositional features (Figure 4, Sections A, B and C). The SEM analysis indicates notable fractures in proximity to oxides identified as lepidocrocite and magnetite. In the upper left region, an accumulation of lepidocrocite appears in bird- and worm nest-like formations, accompanied by visible delamination of oxyhydroxide layers from the steel surface (Section A—A1 to A3). This behavior is attributed to the high porosity of this portion of the corrosion product, whose detachment facilitates further degradation of the metallic substrate. The central region exhibits eroded lepidocrocite structures with extensive cracking. These bird’s nest-like and sandy crystal agglomerates resemble porous coral-like bars, consistent with descriptions in the literature.^45,85,89,94^

Goethite (cotton ball and grassy type), commonly reported in the literature.^37^ forms rounded conglomerates predominantly in the upper-right and central regions. At higher magnification, grass-type formations and cotton ball morphologies characteristic of goethite become evident, along with clearer visualization of magnetite crystals. Worm-like lepidocrocite structures also appear near the main fractures. At even higher magnification, grass-like and cotton ball-shaped formations associated with goethite are observed, as well as worm nests and globular structures located toward the bottom and right. Donut-shaped magnetite crystals are discernible near central fractures (Section B—B1 to B3).

A second set of microstructural observations supports these findings, revealing distinct oxide and oxyhydroxide formations across upper, central, and lower regions of the surface. The upper region shows pronounced exfoliation due to oxide-layer detachment after immersion. Fissures occur near porous features such as acicular, needle-like, wormhole-like, and nest-like morphologies typical of lepidocrocite. At the upper boundary, conglomerates of cotton ball-like goethite and globular lepidocrocite are also observed, extending through the central and lower regions.

At intermediate magnification, grass-like and cotton ball-like goethite conglomerates become evident, along with bird nest and globular morphologies associated with lepidocrocite. At the highest magnification, this oxide and oxyhydroxide structures are confirmed with greater precision. Additional observations reveal combined structures composed of lepidocrocite bird nest-like features interspersed with sandy crystalline formations, forming a porous coating with voids that may facilitate chloride ingress, as previously discussed (Section C—C1 to C3).

The EDS analysis characterizes the elemental composition of the sample treated with PIL 02 after immersion. The introduction of PIL 02 alters the surface composition: iron content exceeds 50%, indicating enhanced formation of iron oxides or hydroxides, while oxygen decreases to 6.84%, consistent with transformations associated with lepidocrocite, goethite, and magnetite formation. Nitrogen increases to 1.82%, attributed to the amine groups in the inhibitor’s structure, whose only difference from PIL 01 is the carbon backbone of the precursor.

Carbon content decreases to 0.33%, whereas chloride increases to 2.49%, aligning with the presence of lepidocrocite, especially along surface regions where chloride adsorption is favored, as detailed in Figure 5. Sodium levels remain relatively stable but appear lower due to preferential chloride retention. The Fe signal reveals heterogeneous concentrations, and differences between PILs 01 and 02 indicate distinct film uniformity and thickness, with PIL 02 showing more discontinuities. The oxygen, nitrogen, and sodium signals display similar distributions, marked by voids consistent with spatially variable oxide formation.

Chlorine is widely distributed, with higher accumulation in specific regions, characteristic of permeable lepidocrocite. This behavior contrasts with the denser goethite phase, which provides more effective barrier properties. Nitrogen shows widespread distribution with voids potentially associated with oxide-rich regions, while carbon remains limited relative to other elements.

The analysis of lepidocrocite is essential due to its relevance as an indicator of chloride accumulation and corrosion progression. Lepidocrocite readily forms in chloride-rich environments and can transform into maghemite, compromising material stability. Its presence affects surface permeability and mechanical integrity, contributing to degradation in aggressive environments and influencing interactions with other ionic species. Understanding these processes is critical for corrosion mitigation and for environmental monitoring in contaminated or coastal regions, details Figure 5.

## 4 Elemental Analysis by Mapping the Rust Section from Microscopic Techniques

Energy Dispersive Spectroscopy (EDS) mapping is a fundamental technique for characterizing corrosion products, as it enables the identification and spatial distribution of key elements such as iron, oxygen, and carbon on corroded surfaces. This technique provides insights into the composition and microstructural features of corrosion products, including the presence of oxides, hydroxides, and porosities, all of which significantly influence the integrity of metallic substrates.^95,96^

In addition, to offering qualitative and quantitative information regarding the composition of corrosion products, EDS allows the correlation of these data with the surface morphology, supporting the development of effective corrosion mitigation strategies. According to the literature, EDS provides essential insights for selecting protective coatings, corrosion inhibitors, and surface treatments, thereby contributing to enhanced material durability in aggressive environments.^97,98^ In the blank system, where the steel sample was immersed in saline solution without corrosion inhibitors, EDS mapping reveals key features related to the corrosion process and the development of corrosion products.

The elemental mapping results also provide important evidence regarding the role of the protic ionic liquids in modifying the corrosion process. Since all samples were exposed to the same saline electrolyte, the differences observed in elemental distribution cannot be attributed to variations in chloride concentration or other environmental parameters. Instead, they reflect the influence of the inhibitors on the formation, composition, and spatial organization of the corrosion products formed on the steel surface.

In the uninhibited system, the widespread distribution of chloride ions across the corrosion layer indicates significant permeability of the oxide film, which is consistent with the presence of porous phases such as lepidocrocite. These phases typically exhibit a lamellar or platelet-like structure that facilitates the diffusion of aggressive species toward the metal surface, thereby sustaining the corrosion process.

In contrast, the systems containing PIL inhibitors exhibit notable differences in elemental distribution, particularly near the metal–oxide interface. The formation of thicker corrosion product layers enriched in iron and oxygen suggests the development of more compact oxide/oxyhydroxide structures. These results are consistent with the identification of goethite in the inhibited samples.

Goethite is widely recognized as a denser and more stable corrosion product, often forming inner layers with lower porosity compared with metastable oxyhydroxides. The presence of such compact structures can significantly reduce the transport of aggressive species, including chloride ions, through the corrosion layer. Therefore, the EDS mapping results support the hypothesis that the inhibitors do not simply reduce the corrosion rate but also influence the structural evolution of the corrosion products, promoting the formation of phases that contribute to improved barrier properties and enhanced corrosion resistance.

A substantial layer of residues forms between the Bakelite mounting material and the carbon steel surface, indicating significant accumulation capable of progressively isolating the metal from the external environment. This layer displays notable porosity, evidenced by voids linked to the formation of oxides and oxyhydroxides. These observations align with previous line scan data, which showed oscillations in the concentrations of O, Fe, N, C, Na, and Cl. The porous structure indicates marked structural alterations, increasing permeability and compromising the protective efficiency of the corrosion layer, as detailed in Figure 6 in section A.

**Figure 6:**
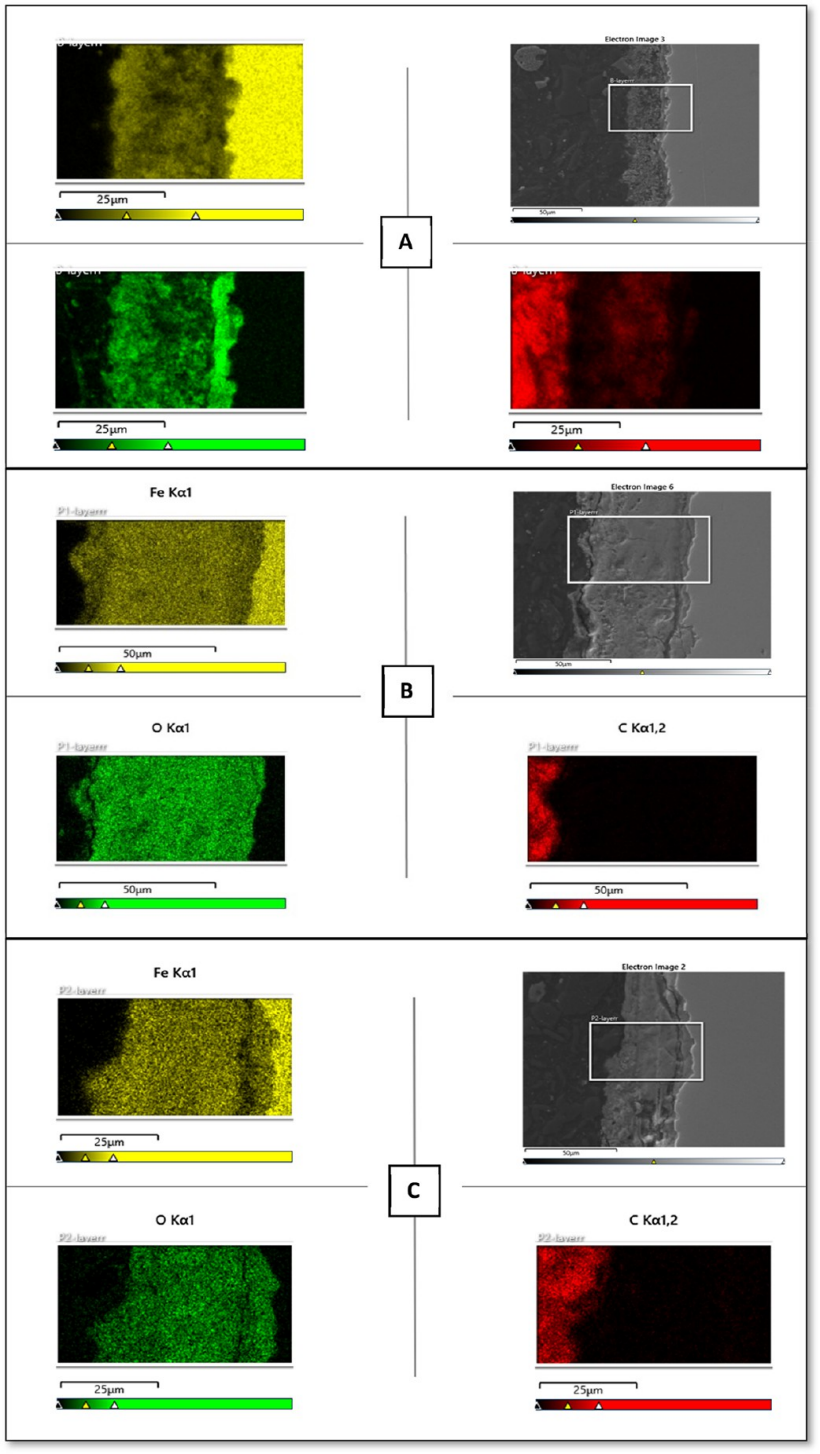
Scanning electron microscopy (SEM) images and energy dispersive spectroscopy (EDS) mapping of carbon steel surface after 24 h immersion without the PILs (A—Blank, B—PIL 01, C—PIL 02).

The distribution of iron shows multiple discontinuities across the steel surface, attributed to the porosity of the corrosion layer and to variations in contrast that reflect morphological degradation. After 24 h of immersion in 3.5 wt % NaCl, the steel surface exhibits pronounced deterioration, particularly because the absence of inhibitors leaves the material fully exposed to the aggressive saline environment.

The oxygen distribution indicates numerous voids within the corrosion layer, reinforcing its elevated permeability and revealing sharper concentration gradients near the steel substrate. These features may relate to magnetite formation in this region, where oxygen accumulates more locally, consistent with previous reports.^1,37,46,62^

Carbon distribution highlights two main regions: the Bakelite matrix and the porous corrosion layer, suggesting that the degraded structure allows carbon diffusion across the surface. Overall, the blank system displays high porosity and heterogeneous elemental distribution, which facilitates the penetration of chloride ions and accelerates material degradation, a behavior also reported in the literature.^37^ When the corrosion inhibitor PIL 01, based on ethanolamine, is introduced into the system, the EDS analysis reveals notable differences in the formation and distribution of corrosion products.

A dense, compact layer is formed between the Bakelite and the steel substrate, indicating a significant accumulation of corrosion byproducts that act as a barrier between the metal and the external environment. Previous studies emphasize the importance of characterizing corrosion products through techniques such as Raman spectroscopy and XRD, which provide insights into morphological features and degradation mechanisms, allowing more accurate interpretation of corrosion behavior.^45^ Such characterization is essential for identifying phase evolution, environmental effects, and protective strategies.

In the iron mapping, a significant increase in the size of the corrosion product formations is observed compared to the blank system. Measurements indicate that while the blank sample exhibits structures exceeding 50 μm, the addition of PIL 01 results in formations larger than 100 μm. The thicker and denser corrosion layer mitigates the harmful effects of chloride ions and produces a more homogeneous surface. The corrosion product also displays increased density, as seen in the more uniform distribution of iron and the reduced presence of voids or discontinuities.

In the central region, variations in oxide and oxyhydroxide distribution are observed, producing intensity gradients indicative of localized accumulation or the presence of circular void-like structures. Near the steel substrate, the reduced iron intensity suggests the formation of a more compact inner layer, likely associated with goethite (α-FeOOH). This phase is known for its structural compaction and effectiveness in limiting the ingress of chloride ions, enhancing the protective behavior of the corrosion layer, as detailed in Figure 6 in section B.

The oxygen distribution confirms extensive oxide formation across the analyzed region. However, in the outermost portion of the sample, cracks and open voids appear, likely associated with more porous oxidation regions that weaken the protective layer. These discontinuities facilitate the diffusion of aggressive species and highlight the importance of phase composition in corrosion resistance.

The central region contains heterogeneous oxygen concentrations, suggesting the coexistence of multiple oxide and oxyhydroxide phases formed during exposure to the saline medium. Close to the steel substrate, especially toward the right-hand side, a compact zone characterized by goethite becomes evident, forming a dense inner layer. Carbon distribution, originating from the Bakelite matrix, appears mainly near the outer boundary of the cross-section. The substantial thickness of the corrosion product formed in the presence of PIL 01 indicating the inhibitor’s role in promoting a thicker and more protective oxide film. In contrast, the blank sample does not present a similarly developed oxide structure after 24 h of immersion in NaCl solution Figure 6 in section B.

In the system containing PIL 02, based on diethanolamine, the EDS mapping likewise provides valuable insights into corrosion product formation. A considerably thicker layer of corrosion residues is observed, along with a prominent crack near the steel substrate. These characteristics indicate a substantial accumulation of corrosion products that form a physical barrier between the metal and the external environment. Compared with the PIL 01 system, the PIL 02 condition reveals a structure enriched in iron, resembling the distribution observed in the previous inhibitor-treated sample, though with more evident microcracks and localized damage.

These defects may result from mechanical stress or structural instability within the corrosion layer. The behavior can be related to the oxide phases present, lepidocrocite, magnetite, and goethite, which influence the compactness of the corrosion product. Literature indicates that goethite forms denser inner layers;^64,99^ therefore, when this phase is less dominant, less compact phases such as lepidocrocite or magnetite may explain the presence of cracks and discontinuities Figure 6 in section C.

The oxygen mapping follows the trends of the iron distribution but shows slightly higher oxygen levels near the steel substrate compared to the PIL 01 system, where oxygen concentrations in this region were lower. These differences confirm that the oxide phases vary with the inhibitor used, influencing the internal arrangement and overall structural integrity of the corrosion layer. Finally, carbon distribution in the PIL 02 system aligns with previous observations: as corrosion products accumulate and become thicker, the contribution of carbon from the Bakelite decreases, making the carbon signal similar in both inhibitor-treated systems.

## 5 Advanced Microscopic Techniques for Surface Morphology and Elemental Analysis of Corrosion Product Layers

Lastly, the FIB-SEM analysis was employed to investigate the formation of oxidation products (oxides and oxyhydroxides) over the carbon steel sample (ASTM A36) and to evaluate the permeability of the resulting layer on A36 carbon steel following 24 h of immersion in a 3.5 wt % NaCl solution, as illustrated in Figures 7 and 8 after the cleaning/removing step using the FIB ion beam. Practically, in all cases, the surface morphology of the carbon steel after the immersion period clearly indicates the presence of specific corrosion products mentioned in the sections above, which were exposed using focused ion beam (FIB) preparation. In sum, the primary objective of this technique is to generate a cross-sectional opening through the dense and scratched surface layer that formed on the samples.

**Figure 7:**
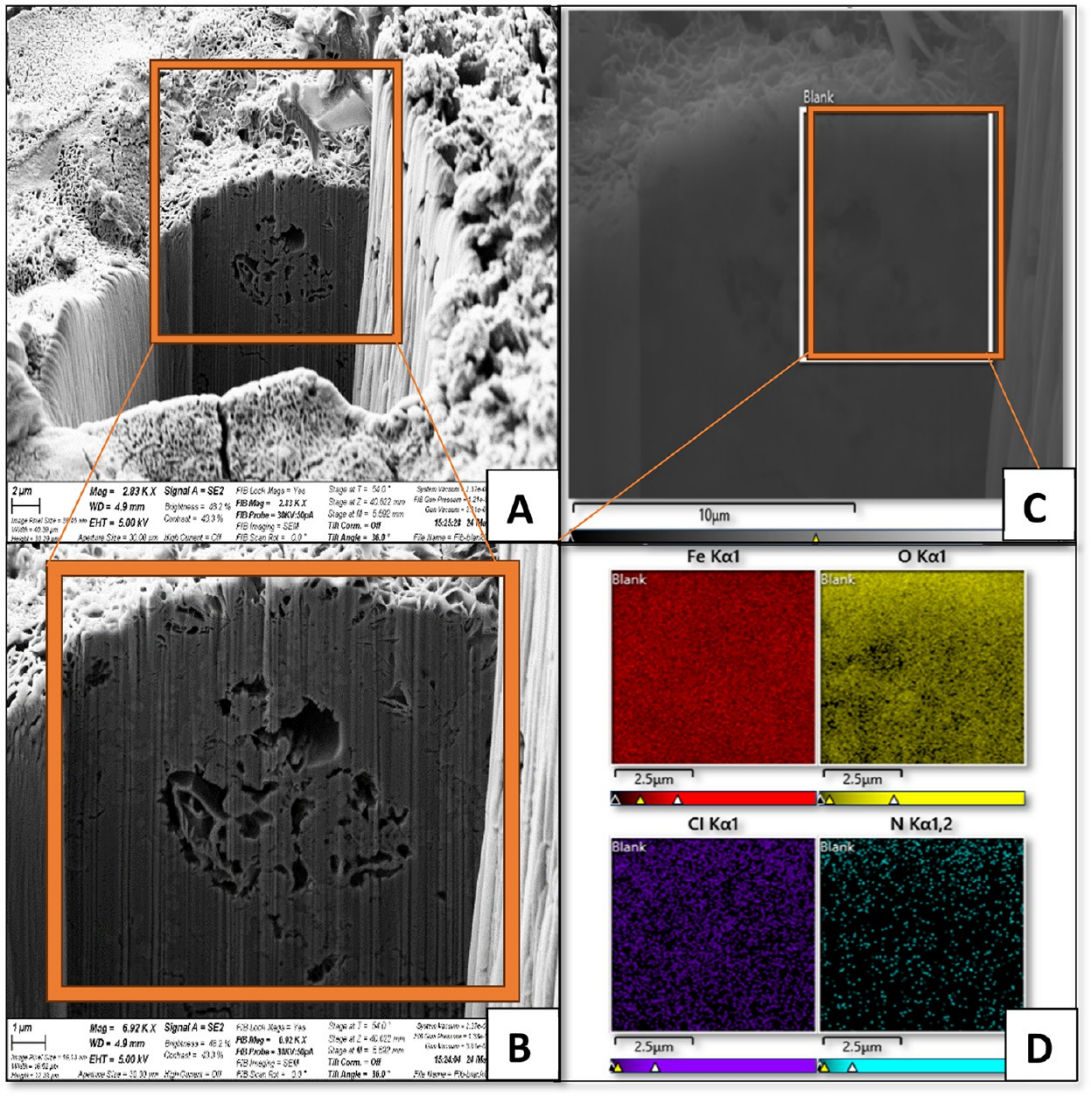
SEM/FIB cross-sectional micrographs and EDX elemental maps (Fe, O, Cl, and N) of the sample exposed to a 3.5 wt % NaCl solution at 25 °C for 24 h without corrosion inhibitors. (A and B) Low- and high-magnification FIB cross-sectional images, respectively, revealing the morphology and thickness of the corrosion layer formed on the steel surface. (C) FIB-sectioned region selected for EDX analysis. (D) Corresponding elemental distribution maps, highlighting the spatial distribution of Fe, O, Cl, and N within the analyzed region.

**Figure 8:**
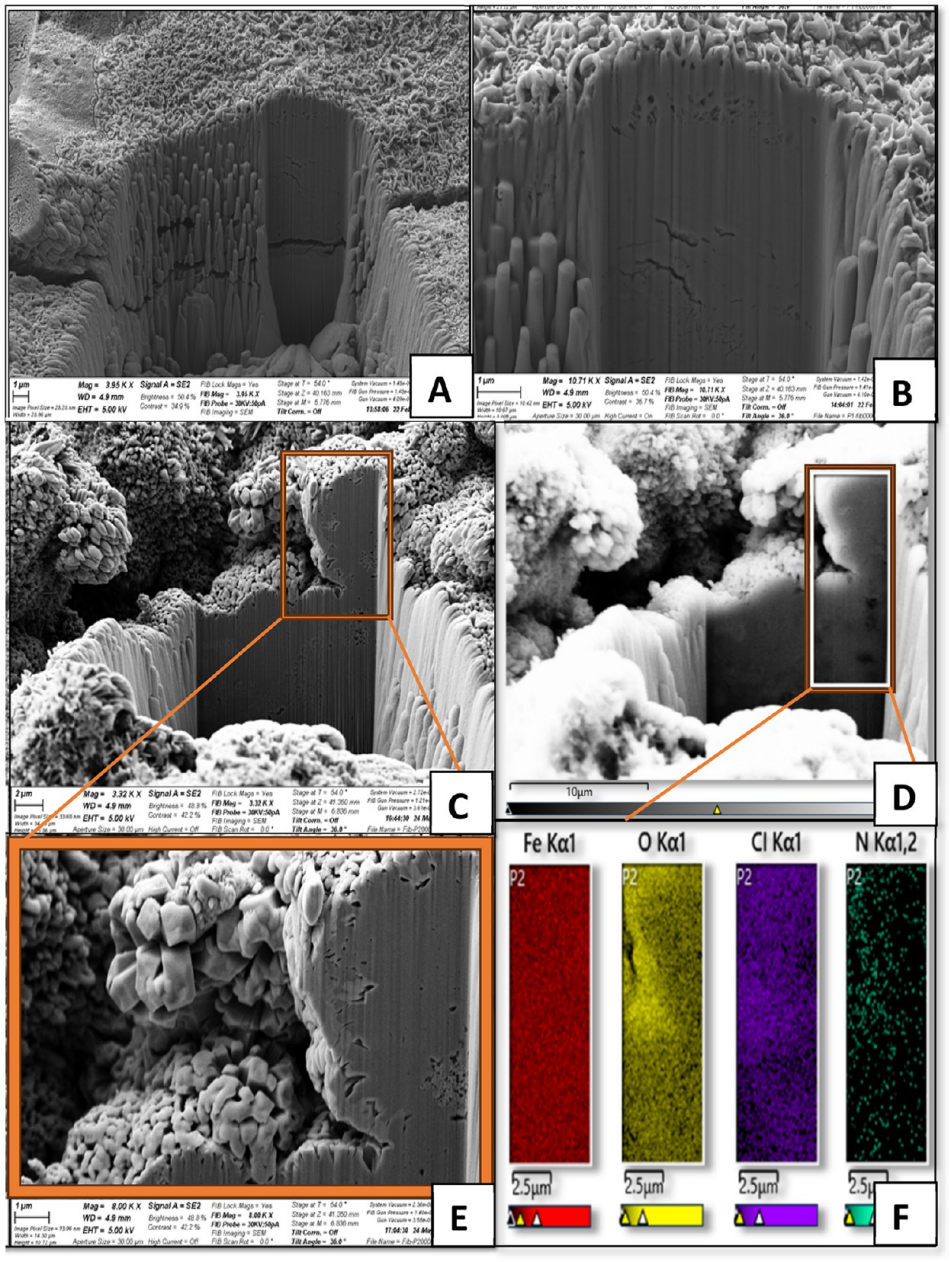
SEM/FIB images of samples exposed for 24 h to saline solutions containing (A, B) PIL 01 and (C–E) PIL 02 at room temperature, along with EDX elemental maps (Fe, O, Cl, N) for the sample treated with PIL 02. The cross-sectioned regions reveal the corrosion layer morphology, while the elemental maps highlight the spatial distribution of the main elements within the analyzed area.

This localized removal allows a more accurate evaluation of subsurface features within a defined region of interest, enabling the assessment of corrosion-layer compactness and the identification of elemental constituents. Such information is essential for determining whether the presence of inhibitors specifically Protic Ionic Liquids (PILs), in the saline solution influences corrosion progression and material degradation over time. Figure 7, organized into four sections (A and B: FIB; C and D: EDS), presents the control sample, corresponding to the system without corrosion inhibitors. In the region containing the phases previously identified by X-ray diffraction, magnetite and lepidocrocite, the corrosion layer appears highly degraded and permeable, evidenced by the visible spacing within the lepidocrocite phase.

Also, a transverse section was created in this area using a localized ion beam, followed by a refinement process, in summary, this cross-section revealed porous structures containing voids and cracks that extended below the surface of the corrosion layer, it is worth noting that these phases arise in the system when it is immersed in saline solution without corrosion inhibitors, resulting in a porous and accessible matrix that remains permeable to ion transport from the solution. In addition, corroborating to earlier EDS mapping analysis results that exposed the access of elements such as chloride, oxygen, and carbon to the steel substrate in the lower region of the examined image.^38,100^

These results are essential for understanding the behavior of systems with and without corrosion inhibitors in saline environments. For samples containing PIL 01 and PIL 02, the formation of goethite, along with other identified phases, produces a denser and more compact layer on the metal surface. This is illustrated in Figure 8, organized into four sections (A and B: FIB—PIL 01; C and E: FIB—PIL 02; D and F: EDS—PIL 02). According to the literature, such a compact layer effectively restricts chloride permeation into the steel, highlighting the long-term protective action of the inhibitors. Additionally, isolated lepidocrocite structures with flake-like and petal-like morphologies were observed, consistent with previous reports on oxide and oxyhydroxide characterization.^38,100,101^

Ongoing, to assist the FIB discussion/analysis, Energy Dispersive X-ray Spectroscopy (EDS) as mapping mode was employed to evaluate a selected region and assess the distribution of chemical elements (Fe, O, N, Cl). Basically, the objective of examining this external region characterized by voids within the oxide/oxyhydroxide layer was to obtain a more comprehensive understanding of the permeation mechanisms occurring between the layers of corrosion products on the steel surface following immersion.

Iron, the first element analyzed and represented in red, was observed throughout most of the scanned area. This widespread presence is attributed to the role of iron as the principal constituent in oxide/hydroxide complexes, resulting in its high surface concentration. Notably, in the sample referred to as the “blank” which lacked corrosion inhibitors, distinct regions with variable iron concentrations were identified. This variation is evident in the form of darker zones in the image, corresponding to areas of reduced iron concentration due to intensity differences. One plausible explanation for this distribution is the formation of iron oxyhydroxides, particularly lepidocrocite, within the outer layer. As reported in the literature, lepidocrocite commonly forms a structure resembling a bird’s nest, characterized by numerous vacancies.

This structural morphology promotes void formation, resulting in a nonuniform and less compact accumulation of corrosion products over time. The subsequent analysis examined the distribution of oxygen (yellow), revealing a pronounced presence across the surface, consistent with the iron results. This indicates the oxygen content within the identified oxides and oxyhydroxides. A higher oxygen concentration appeared near the interface exposed to the saline solution, likely due to its high permeability, which enables continuous oxygen progress from the external environment.

Further, a subsurface layer also exhibited a notable oxygen signal, though interspersed with voids. This heterogeneity may be associated with localized iron accumulation, which creates unoccupied regions within the corrosion product layer. In the absence of an inhibitor, the formation of a dense and compact goethite layer was not observed in this blank sample, leading to a corrosion product layer with pronounced porosity.^38^ Furthermore, in the innermost region adjacent to the carbon steel matrix, a similar increase in spacing was observed, consistent with the pattern identified in the central region. This behavior can probably be attributed to the absence of a well-defined goethite oxyhydroxide phase in the blank sample.

This likely results from pH differences between inhibited and uninhibited solutions: the blank remains neutral/slightly acidic, while PILs render the medium alkaline. Since goethite forms a compact inner barrier layer, the oxide formed here likely exhibits incomplete structural filling under the present experimental conditions. Chloride ions represent a valuable factor in the assessment of corrosion in saline media. The literature consistently reports that when a protective film fails to effectively prevent the permeation of such aggressive ions, the overall efficacy of the corrosion inhibitor is significantly diminished. In the present study, the oxides and oxyhydroxides identified suggest that the formation of a specific phase could contribute to the development of a compact barrier, capable of impeding chloride ion diffusion through the corrosion products and thereby protecting the underlying metal substrate. Energy-dispersive X-ray spectroscopy (EDS) analysis of chloride distribution revealed a more widespread dispersion of chloride ions in the region analyzed. This finding confirms that the corrosion product formed under these conditions is insufficient to effectively limit chloride permeation.

Nitrogen also plays an important role in EDS analysis. Since PILs 01 and 02 contain amine-based functional groups (ethanolamine and diethanolamine), nitrogen accumulation in the corrosion products may offer insights into the characteristics of the formed layer. In the blank sample (Figure 7), nitrogen appears more dispersed and at much lower concentrations than Fe, O, and Cl. Its presence may be linked to the acidic electrolyte, which can generate nitrate (NO_3_^–^) and ammonium (NH_4_^+^). These permeable species interact with Fe^2+^ and Fe^3+^ during corrosion, promoting nitrogen incorporation within the corrosion products on the carbon steel surface. The surface analysis revealed that the formation of lepidocrocite, goethite, and magnetite was discovered. The phases of lepidocrocite oxyhydroxide were identified in distinct morphologies. The “worm nest” structures were observed in the central region of the sample^45,85^ while the “flakes” morphology appeared in the upper right region,^102^ and “sandy crystals” were found on the left side of the image.^45,94,99^ In addition, Magnetite was identified based on its morphology, characterized by “fine spherical oxides” located in the upper central region.^32^ In sum, these phases were recognized according to morphologies documented in previous studies on corrosion products. The formation of goethite oxyhydroxide was characterized by the presence of “lenticular crystals” in the central region^45,85,102,103^ and “cotton balls” in the left central area.^45,46,62,85,90,104^ Acicular goethite (filiform) was also observed in the sample.^45^ In this context, identifying these phases was based on the nomenclature and morphological classifications reported in corrosion literature.

The evaluation of the oxidized region after a period of immersion in solution, where oxides and oxyhydroxides were identified along with the accumulation of corrosion products. A dense and compact layer was observed after sectioning with the aid of the FIB technique. As shown in Figure 8, sections A, B, C, and E, this compact and dense layer notably differs from what was observed in evaluating the blank sample. It is important to note that a crack was identified in this section, and the likely cause of this failure is the presence of magnetite and lepidocrocite surrounding the selected region.

The presence of these iron oxide/oxyhydroxide phases is linked to potential permeability, and where these voids exist, the possibility of failure increases. The evaluation of corrosion products is crucial to understanding how the protective film, initiated by the addition of the corrosion inhibitor in the system, will evolve over time. In this study, a 24-h period was used as the base, and the formation of the corrosion product layer was sufficient for the analysis using the FIB technique.

Continuing the evaluation of the carbon steel surface after immersion in saline solution with the addition of PIL 02 as a corrosion inhibitor, the following are the primary oxides/oxyhydroxides and their respective morphology. In section B, the formation of magnetite (Fe_3_O_4_) was observed, characterized by the morphology of “fine spherical oxides” located in the upper central area of the steel surface. Additionally, other morphologies were identified, including “flake structures” in the right region and “sandy crystals” in the left region of the image. This identification was based on a comprehensive literature review on corrosion products, and the results were confirmed through characterization techniques and electron microscopy.^32,37,38,85,100^

In addition to the identification of corrosion products, lepidocrocite oxyhydroxide was observed on the surface. The morphologies detected for this phase included the accumulation of sandy crystals, specifically located in the upper left region of section B. This accumulation corresponds to the morphology referred to as the “bar type” in literature. Additionally, the “worm” and “bird nest” morphologies, commonly observed in atmospheric corrosion studies in saline environments, were also identified. These were located in the lower left region, near the FIB sectioning area.^37,38,45,85^

Lastly, regarding the corrosion products, the last identified oxyhydroxide was goethite. The identification of goethite is particularly important, as the formation of a denser inner layer can hinder the permeation of ions, such as chloride, into the metallic matrix.

The identified morphologies included the “cotton balls” structure, which was found in the upper right, central, and left regions, covering almost the entire selected area. Additionally, globular and nested formations were observed. These identifications were made based on corrosion product literature.^38,45,46,62,85,90,104^ Continuing the analysis, Figure 8, specifically section B, depicts the evaluation of the steel surface following immersion in a saline solution containing PIL 02 as a corrosion inhibitor. The objective of this analysis was to determine whether corrosion residues had accumulated in the region and to assess their potential impact on the protection of the underlying metallic substrate. The study sought to evaluate whether a denser, more cohesive protective layer would form after exposure of the deeper layers via focused ion beam (FIB) analysis. In section B, a compact layer was observed beneath the agglomerated corrosion products. In contrast to the blank sample, which lacked corrosion inhibitors and exhibited cracks, voids, and discontinuities formed during the immersion period, the presence of this layer suggested a more stable morphology. The primary aim of employing this technique was to investigate whether the formation of a specific iron oxyhydroxide phase, namely, goethite could effectively hinder ionic permeation to the metallic matrix.

In this context, the presence of goethite, as evidenced in section B, is likely associated with a notable reduction in permeability, particularly over extended exposure periods. According to the corrosion literature, variations in pH and temperature play a critical role in promoting goethite formation, as lepidocrocite can dissolve and subsequently precipitate as goethite when the system evolves under alkaline conditions (pH > 7). Prior to immersion, the pH of the blank system was measured at 6.10, whereas both solutions containing PILs exhibited pH values exceeding 8.5 conditions favorable for goethite formation. In conclusion, the characterization of corrosion products is essential for understanding the protective behavior of the inhibitor-induced surface film over time. In the present study, a 24-h immersion period was sufficient to allow for FIB analysis of the corrosion product layer, underscoring the inhibitor’s effectiveness in mitigating corrosion processes.

Continuing the evaluation of the corrosion inhibitors, specifically the PIL 02, the technique applied in parallel with the FIB analysis was Energy Dispersive X-ray Spectroscopy (EDS) (Figure 8—Section D and F), which involved mapping a selection to evaluate the concentration of chemical elements (Fe, O, N, Cl).

In this context, the objective was to analyze the outermost region, which comprises characteristic oxide and oxyhydroxide formations, specifically goethite and magnetite. This evaluation aimed to provide insight into the permeation processes occurring between the corrosion product layers following immersion in a saline solution containing the corrosion inhibitor PIL 02.

The first element assessed was iron, once again represented by the color red. Iron is distributed across nearly the entire image, reflecting its predominant role in the formation of oxide and hydroxide complexes and its consequent high surface concentration. In the control sample referred to as the blank, in which no corrosion inhibitors were present extensive regions exhibiting variable iron concentrations were observed. This behavior is evident in the image through the appearance of darker areas, where variations in intensity indicate lower iron concentrations. In contrast, the sample exposed to PIL 02 exhibited a uniformly higher iron concentration across the entire region, with no discernible areas of fluctuating elemental intensity

Therefore, a possible explanation for this result would be that there is the formation of a large amount of corrosion product, mainly magnetite and goethite, and both phases are characterized by a high iron content in their composition.^38^ In this evaluation, we sought to investigate an area with the highest concentration of cotton balls (goethites) and fine spherical oxides (magnetites) forming a cluster of corrosion products. The objective of this analysis was to determine whether the formation of the surface layer influences the permeation of specific ions to the metal substrate.

Based on the findings obtained through EDS analysis focused on iron evaluation, it was observed that the presence of corrosion inhibitors significantly enhanced the accumulation of this element on the metal surface.

This suggests that the development of the oxide layer serves as a source of fundamental elements, potentially facilitating the permeation of other species, such as chloride ions, as reported in the literature. Subsequently, the distribution of oxygen was examined, as presented in Figure 8. A marked concentration of oxygen was detected at the surface, in contrast to the more uniform distribution observed for iron. This result is attributed to the presence of oxygen in the composition of the oxides and/or oxyhydroxides formed on the steel surface. The objective in this case was to assess the elemental composition of the primary corrosion products identified as cotton ball-like formations (goethite) and fine spherical structures (magnetite).

In the region closest to the surface, which was exposed to the saline solution and an aerated environment, a notably higher concentration of oxygen was detected, attributable to direct exposure. In fact, a particular interest was the observation that this elevated oxygen concentration was predominantly confined to the outermost layer. As the analysis progressed toward the interior of the corrosion product, a gradual decrease in oxygen content was observed. This trend can be attributed to the high density of the corrosion layer, which inhibits the continuous diffusion of oxygen from the external environment and the solution into the internal regions.

Furthermore, chlorine was analyzed as a key element in understanding chloride ion behavior. Its presence is a critical factor in evaluating the degradation experienced by steel under aggressive saline conditions. Specifically, the chloride ion (Cl^–^), present in NaCl solutions, is highly corrosive to carbon steel as it penetrates and destabilizes the passive oxide layer, thereby exposing the underlying metal to further corrosion. Additionally, chloride ions enhance the conductivity of the medium, thereby facilitating electrochemical reactions that accelerate the overall corrosion process.^105^

The chloride ion also facilitates the formation of galvanic cells on heterogeneous metallic surfaces, thereby intensifying localized oxidation in specific regions of the metal. A further detrimental effect of chloride is its propensity to induce localized corrosion, particularly pitting corrosion, which results in the formation of small cavities on the steel surface and may ultimately compromise structural integrity.

Additionally, chloride ions can react with corrosion products to form soluble complexes that disrupt and remove protective oxide layers, thereby sustaining the corrosive process. Collectively, these phenomena establish chloride as one of the principal agents responsible for the accelerated degradation of carbon steel in saline environments.^105^ As observed in the blank sample, the literature consistently emphasizes that when the protective film formed by the chemical constituents fails to prevent the permeation of highly aggressive ions, the effectiveness of the corrosion inhibitor is significantly diminished.

Accordingly, in the present study involving the addition of inhibitors, particularly illustrated in Figure 8, the oxides and oxyhydroxides identified suggest that the formation of a specific phase, namely goethite, contributes to the development of a compact barrier.

This barrier effectively hinders the diffusion of chloride ions through the corrosion product, thereby providing sustained protection to the metal substrate over time. When evaluating the system with the addition of corrosion inhibitors, it was concluded from the energy dispersive X-ray spectroscopy (EDS) analysis that the chloride distribution presented a more diffuse pattern in the outermost area than when compared to the interior of the selected region that was at the border of the corrosion product.

Therefore, this finding confirms that the observed corrosion product is indeed capable of effectively restricting the permeation of chloride ions with the addition of corrosion inhibitors after a certain immersion period, which in this case was 24 h. Furthermore, as a result, over time, these ions will not penetrate more extensively, leading to less significant degradation of the metal matrix as was found from the other techniques already discussed in other topics previously. Finally, nitrogen can be considered the crucial element to be evaluated using the energy dispersive X-ray spectroscopy (EDS) analysis technique. The reason is quite trivial when studying the influence of corrosion inhibitors, in this case protic ionic liquids (PILs 01 and 02).

Because these compounds contain in their composition the presence of amines located in the primary basic fraction (ethanolamine and diethanolamine), and the accumulation of nitrogen in the corrosion product agglomerate is a very important result for the permeability evaluation. As illustrated in Figure 7 for the blank sample without addition of corrosion inhibitors, the distribution of the nitrogen element is much more dispersed in the corrosion product and considerably lower when compared to the other elements. Therefore, in contrast to the blank system, where minimal nitrogen detection was observed, the high nitrogen content in the outermost region of the corrosion product formed when carbon steel (A36) was exposed to the electrolytic medium (3.5% NaCl) with the addition of corrosion inhibitors can be attributed to the presence of amines in the formulation of these compounds.

Amines play a crucial role in the formation of the protective film, which results from the interaction between the inhibitor molecules and the steel surface. This interaction may also persist over time. As the exposure period extends beyond 24 h, the outermost layer of the corrosion product contains a higher nitrogen concentration. According to the literature, this phenomenon can be attributed to the increasing nitrogen content in the composition of the corrosive film, which enhances the stability of the protective layer, prevents chloride ion penetration, and improves the film’s compaction.^106^

## 6 Discussion

Therefore, based on the experimental results described in the previous topics, Figure 9 seeks to describe in a didactic way how the process of formation and evolution of rust layers occurs on the surface of carbon steel when evaluated in a saline medium (3.5% by weight) at room temperature with and without the addition of corrosion inhibitors. Thus, as shown in 9, the initial corrosion products illustrated with yellow coloration (Goethite - α-FeO­(OH)), orange filled and as a network (Lepidocrocite γ-FeO­(OH)) and brown (Magnetite - Fe_3_O_4_) began to accumulate on the steel substrate.

**Figure 9:**
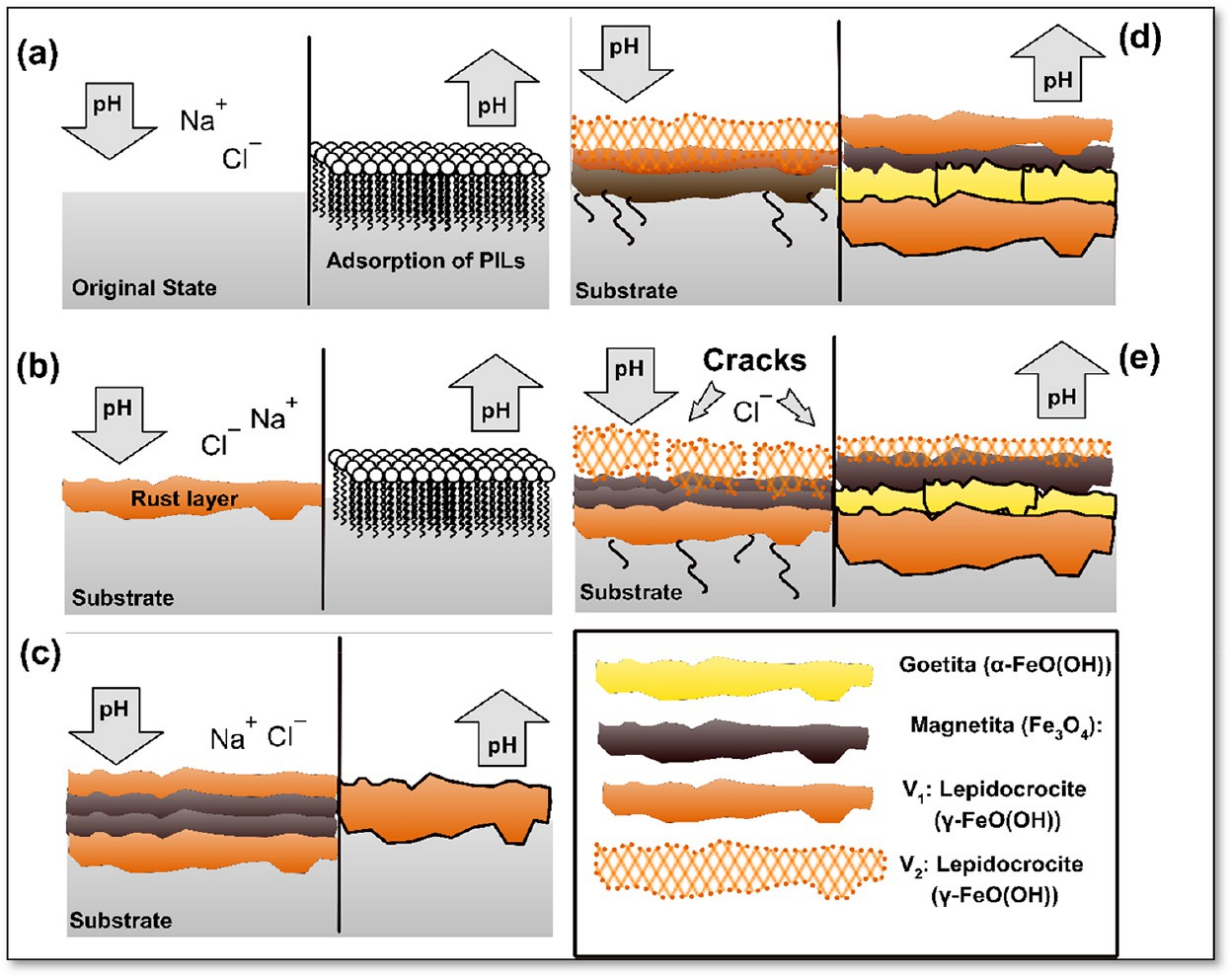
Diagram illustrating the formation and progression of the rust layer (comprising oxides and oxyhydroxides) during saline corrosion inhibitors (PILs).

Initially, in order to contextualize the sequence of events that are portrayed in the image, there is the addition of the corrosion inhibitor in a saline solution at a certain concentration. Right in section (a), there are 2 distinct regions where we sought to simulate a sample that was subjected to the medium with the addition of the inhibitor and without its presence. This differentiation was made with the indication of the pH because in the electrolytic medium where the best corrosion inhibitors were added (PILs 01 and 02), the pH value was slightly alkaline, different from that found in the blank without their addition.

Thus, in section (a), it is possible to observe that, due to the absorption of corrosion inhibitors, a protective layer is formed, which is different from what is observed on the left, where there is a direct attack of the chloride ion on the steel surface. As the exposure time increases, the initial corrosion products close to the substrate gradually transform into components (oxyhydroxides), in this specific case lepidocrocite (section b), which is represented in orange in its first version (V_1_), which is more stable and intact, different from the second version (V_2_), where there is a corrosion product containing interlaces and spaces.

It was decided to choose two distinct versions for this type of oxyhydroxide, because over time it presents permeations in the form of a network containing spaces and ends up becoming brittle, which can cause cracks over time. Continuing, in section (c), it is possible to observe the evolution of this corrosion product formation occurring in parallel with the greater wear of the carbon steel surface as this layer evolves in thickness. Thus, what is seen on the left in this section, without the addition of inhibitors, is the formation of a more extensive layer containing other phases of iron oxides.

Thus, in this region, the formation of a more internal layer of Magnetite (Fe_3_O_4_), represented by the brown coloration after the evolution of Lepidocrocite that was already represented in the previous section, is observed. On the right, in section (c), there is the formation of a thicker layer of Lepidocrocite (V_1_) being formed after the adsorption stages of the corrosion inhibitors. This phenomenon can be attributed to the fact that the adsorption of the film on the steel surface slows down the degradation of the surface, creating a layer of corrosion product gradually.

Still on Figure 9, in section (d), there is a passage of time during this immersion period, possibly 24 h as evaluated in the immersion tests discussed previously. In the left area, where the presence of inhibitors in solution was not related, there is much greater wear in relation to the steel surface. This result is evidenced first by the irregularities found below the corrosion product and the thin thickness of the final corrosion product formed on the steel surface. Furthermore, on the right of section (d), one can observe the formation of all corrosion products identified in the study, such as lepidocrocite, magnetite, and goethite on the steel surface.

In detail, what is crucial to observe about this arrangement of oxides/oxyhydroxides is that there are formations that are more internal to the steel surface and more external, highlighting the denser and more compact formation of goethite (in yellow). Finally, there is the last section (e) addressed in Figure 9. In the left area, the difference observed would be the modification of the lepidocrocite formation, in orange, going from version 1 to 2.

This modification was chosen as an analogy for the high permeation that this oxyhydroxide formation presents over time and its characteristic morphology (bird and worm nest) that presents this interlacing that facilitates the permeation of the chloride ion, accessing the surface of the metal, and that subsequently leads to the appearance of flaws in this rust coating.

On the right of section (e), the only difference is the modification of the lepidocrocite versions as explained previously, however the only difference would be this influence on the chloride permeation because as there is the accumulation of other denser and more compact phases, the aggressive ion does not affect the surface of the carbon steel as much at the end of the exposure period.

An important aspect observed in our study is that the addition of the PILs modified the chemical environment of the solution, particularly by increasing its alkalinity. Measurements of pH and electrical conductivity confirmed that the solutions containing PILs exhibited more alkaline conditions compared to the blank solution. Under alkaline environments, the oxidation of Fe^2+^ to Fe^3+^ and the subsequent formation of Fe­(OH)_3_ can be favored, which may promote the transformation into goethite (α-FeOOH). Although the Mössbauer spectrum showed a more evident signature of goethite for sample PIL02, the presence of this phase was also supported by complementary characterization techniques.

Specifically, Raman spectroscopy, X-ray diffraction (XRD), and advanced microscopy analyses (SEM and FIB) indicated the formation of goethite in samples containing the PIL inhibitors, particularly for PIL01 and PIL02, which were the systems that showed the most pronounced modification in the corrosion product morphology and composition. These results suggest that the formation of goethite is likely associated with the changes in the local chemical environment induced by the PILs, especially the increase in pH that favors the precipitation and stabilization of Fe^3+^ oxyhydroxides.

## 7 Conclusions

This study demonstrates that the protic ionic liquids PIL 01 (2-HEAF) and PIL 02 (2-HDEAF) significantly influence the nature and stability of corrosion products formed on carbon steel in a saline environment. Rather than merely reducing corrosion rates, these inhibitors actively modify the corrosion pathways by altering the oxide/oxyhydroxide phase assemblage developed during exposure.

An important aspect of the present study is that the observed differences in corrosion product composition were not associated with variations in environmental parameters, since all experiments were conducted under identical saline conditions (3.5 wt % NaCl). This controlled experimental design ensures that the changes identified in oxide and oxyhydroxide phases are primarily related to the presence of the protic ionic liquids and their interaction with the carbon steel surface.

In the absence of inhibitors, the corrosion process was dominated by the formation of lepidocrocite and magnetite, resulting in porous corrosion layers that facilitate chloride penetration and sustain electrochemical degradation. Conversely, the addition of PIL inhibitors promoted the development of more compact corrosion product layers, with significant contributions from goethite.

The formation of goethite is particularly relevant because this phase is known to form dense inner layers that reduce the permeability of corrosion products and limit the transport of aggressive species toward the metal surface. As a result, the protective behavior observed in inhibited systems arises not only from the adsorption of inhibitor molecules but also from the modification of corrosion product evolution. This combined mechanism highlights the dual role of the PILs investigated in this work, which involves both electrochemical inhibition and structural stabilization of the corrosion layer.

The uninhibited system is characterized by the predominance of lepidocrocite, resulting in a porous and poorly protective corrosion layer that facilitates chloride penetration. In contrast, the presence of PILs promotes the formation of magnetite and goethite, leading to denser, more compact, and structurally stable corrosion product layers with enhanced barrier properties.

Phase identification by XRD and Mössbauer spectroscopy confirms these transformations, while Raman spectroscopy reveals sharper and better-defined spectral features in inhibited systems, indicating improved crystallinity and phase stability. These results demonstrate a clear concentration- and inhibitor-dependent effect on corrosion product evolution. Morphological analyses using OM, SEM, and FIB corroborate the spectroscopic findings.

Uninhibited samples exhibit severe surface degradation, extensive localized corrosion, and highly porous rust layers, whereas PIL-treated surfaces display reduced porosity, more uniform corrosion layers, and increased layer thickness, consistent with improved protection against chloride ingress. EDS analysis further supports these observations by revealing higher Fe/O ratios and the presence of carbon- and nitrogen-containing species associated with PIL adsorption.

An important outcome of this study is that the addition of protic ionic liquids (PILs) alters the chemical environment of the solution, particularly by increasing its alkalinity. Measurements of pH and electrical conductivity confirmed that PIL-containing systems are more alkaline than the blank solution. Under these conditions, the oxidation of Fe^2+^ to Fe^3+^ and the subsequent formation of Fe­(OH)_3_ are favored, promoting its transformation into goethite (α-FeOOH). Mössbauer spectroscopy revealed a more pronounced signature of goethite for the PIL02 sample; however, the presence of this phase was corroborated by complementary techniques.

Raman spectroscopy, X-ray diffraction (XRD), and microscopy analyses (SEM and FIB) consistently indicated the formation of goethite in PIL-containing systems, particularly for PIL01 and PIL02, which exhibited the most notable changes in corrosion product morphology and composition. These results demonstrate that the formation of goethite is closely linked to the modification of the local chemical environment induced by the PILs, especially the increase in pH, which favors the precipitation and stabilization of Fe^3+^ oxyhydroxides.

Overall, the findings confirm that PILs mitigate corrosion through a dual mechanism: modification of the electrochemical environment at the metal–solution interface and stabilization of protective oxide/oxyhydroxide phases. By emphasizing corrosion product characterization an approach still underexplored in corrosion inhibitor studies this work provides a mechanistically grounded contribution to the understanding of PIL-based inhibition.

These insights highlight the potential of PILs as effective and sustainable corrosion inhibitors for carbon steel in saline environments and establish a foundation for future optimization of inhibitor structure and long-term performance evaluation.

## Supplementary Material



## Data Availability

The original contributions presented in the study are included in the article/Supporting Information; further inquiries can be directed to the corresponding authors.

## Notes

Mössbauer spectroscopy data for the identification and quantification of iron oxides and oxyhydroxides formed on A36 carbon steel in saline media with and without protic ionic liquids (PILs), supported by X-ray diffraction and Raman spectroscopy; hyperfine parameter tables and reference data used for phase assignment; optical microscopy cross-sectional analyses of corrosion product morphology and layer thickness; quantitative measurements of oxide/oxyhydroxide layer development under inhibited and uninhibited conditions; energy- dispersive spectroscopy (EDS) line-scan profiles showing the distribution of Fe, O, Cl, C, and N across the corrosion layers; supplementary figures and tables supporting the correlation between corrosion product composition, chloride penetration, and inhibitor performance discussed in the main manuscript (PDF)
