# Supplementary material for: Assessment of Corrosion Product Formation (Iron Oxides and Oxyhydroxides) on Carbon Steel in Saline Media Containing Protic Ionic Liquids by Advanced Structural and Microstructural Characterization
Source: ACS Omega. 2026 Jun 3;11(23):34199–225. doi: 10.1021/acsomega.6c01592 (PMC13280892; doi:10.1021/acsomega.6c01592)
Supplement: Supplementary file 1 [file ao6c01592_si_001.pdf]

SUPPORTING INFORMATIONS (SI)

Assessment of Corrosion Product Formation (Iron Oxides and Oxyhydroxides) on Carbon Steel in Saline Media Containing Protic Ionic Liquids by Advanced Structural and Microstructural Characterization.

Caio Victor Pereira Pascoal[1]*, Daniel de Castro Girão[1], Bruno Gomes Linhares[1], Samuel Lucas Santos Medeiros[1], Regiane Silva Pinheiro[3], Seyed Ali Razavi[4,5,6], Mohammad Rezayat[4,5,6], Igor Frota de Vasconcelos[1], Hosiberto Batista de Sant'Ana[3], Gemma Fargas [4,5,6], Walney Silva Araújo[1]**.

Assessment of Corrosion Product Formation (Iron Oxides and Oxyhydroxides) on Carbon Steel in Saline Media Containing Protic Ionic Liquids by Advanced Structural and Microstructural Characterization.

Caio Victor Pereira Pascoal[1]*, Daniel de Castro Girão[1], Bruno Gomes Linhares[1], Samuel Lucas Santos Medeiros[1], Regiane Silva Pinheiro[3], Seyed Ali Razavi[4,5,6], Mohammad Rezayat[4,5,6], Igor Frota de Vasconcelos[1], Hosiberto Batista de Sant'Ana[3], Gemma Fargas [4,5,6], Walney Silva Araújo[1]**.

1- Department of Metallurgical and Materials Engineering, Federal University of Ceará, 60440-900 Fortaleza, CE, Brazil.
2- Department of Food Engineering Federal University of Maranhão (UFMA), Imperatriz, Ma, 65915-060, Brazil
3- Department of Chemical Engineering, Federal University of Ceará Fortaleza (UFC), CE, 60440-554, Brazil
4- Center for Structural Integrity, Micromechanics, and Reliability of Materials (CIEFMA)-Department of Materials Science and Engineering, Universitat Politècnica de Catalunya-Barcelona TECH, 08019 Barcelona, Spain.
5- Department of Materials Science and Engineering, EEBE, Universitat Politècnica de Catalunya, UPC, C/Eduard Maristany, 10-14, 08019 Barcelona, Spain
6- Barcelona Research Center in Multiscale Science and Engineering, Universitat Politècnica de Catalunya, UPC, C/Eduard Maristany, 10-14, 08019 Barcelona, Spain.

*Corresponding Authors / Email address: caiovictorppascoal@gmail.com (Caio Victor Pereira Pascoal) / wsa@ufc.br (Walney Silva Araújo) /

Abstract

This study investigates protic ionic liquids (PILs) as corrosion inhibitors for carbon steel in a saline environment, focusing on their influence on the formation and stability of corrosion products during exposure. By examining corrosion product evolution under inhibited conditions, this work provides a mechanistic perspective beyond conventional inhibition efficiency, evaluating whether the resulting corrosion layers can act as a protective barrier during prolonged exposure. X-ray diffraction, Mossbauer, and Raman spectroscopy techniques were applied to characterize the surface and corrosion product. Moreover, optical and scanning electron microscopy techniques (SEM–ED and FIB–SEM) were selected to identify the oxides/oxyhydroxides externally. At the same time, X-ray spectrometers with energy dispersion were used to analyze the elemental composition, mainly O, Fe, N, Cl, and Na. Fundamentally, this preliminary investigation seeks to identify corrosion products (iron oxides/oxyhydroxides), including lepidocrocite, magnetite, and goethite, elucidate their formation mechanisms, and contribute a novel perspective to the corrosion inhibitor literature. In particular, the study focuses on the interaction between carbon steel and Protic Ionic Liquids (PILs), exploring their potential influence on the formation and evolution of distinct oxide phases on the material's surface. As a key distinguishing feature, the addition of the corrosion inhibitors PILs (2-HEAF) and (2-HDEAF) revealed the presence of a dense and compact goethite, a phase that can aid in blocking chloride penetration, potentially extending the longevity of carbon steel in saline environments. This observation supports the efficacy of these inhibitors, as previously demonstrated in electrochemical and weight-loss experiments, suggesting their potential for long-term corrosion protection.

Keywords: A. Protic Ionic Liquids; B. Corrosion Inhibitor; C. Mild Steel A36; D. Oxides and oxyhydroxides.

1. Characterization of corrosion products of A36 carbon stee

1.1 Mossbauer characterization of iron oxides and (oxy)hydroxides.

This section addresses the characterization of corrosion products formed on carbon steel surfaces exposed to saline media in the presence of different Protic Ionic Liquids (PILs). The objective is to assess the influence of PIL chemical structure on the nature, distribution, and stability of iron oxides and oxyhydroxides generated during the corrosion process. Such understanding is essential to correlate molecular features with the formation of protective corrosion layers and to support the development of effective and sustainable corrosion mitigation strategies.

A reliable identification of corrosion products requires the combined application of complementary analytical techniques. In this study, Mössbauer spectroscopy was employed as a primary tool due to its sensitivity to iron-bearing phases and its ability to provide detailed information on their magnetic and electronic environments. Raman spectroscopy and X-ray diffraction (XRD) were used to support phase assignment and to evaluate crystallinity and structural features. The integration of these techniques enables a consistent and robust identification of polymorphic iron oxides and oxyhydroxides formed during exposure to saline environments.

Mössbauer measurements were carried out at room temperature using a SEECo spectrometer (Model W302) equipped with a cobalt-60 source in a rhodium matrix. Spectral fitting was performed using the NORMOS software, applying the site distribution method. Spectra containing magnetic components were analyzed over a Doppler velocity range of ±12 mm s^{-1}. The blank sample, obtained in the absence of inhibitors, consisted exclusively of corrosion product powder and was immediately analyzed.

Its Mössbauer spectrum exhibited one paramagnetic doublet and two magnetic sextets. Based on the extracted hyperfine parameters and supported by XRD data, the doublet was assigned to lepidocrocite (γ-FeOOH), while the sextets were attributed to magnetite (Fe_3O_4), corresponding to tetrahedral and octahedral iron sites. The identification of lepidocrocite is further supported by its characteristic paramagnetic behavior in Mössbauer spectroscopy.

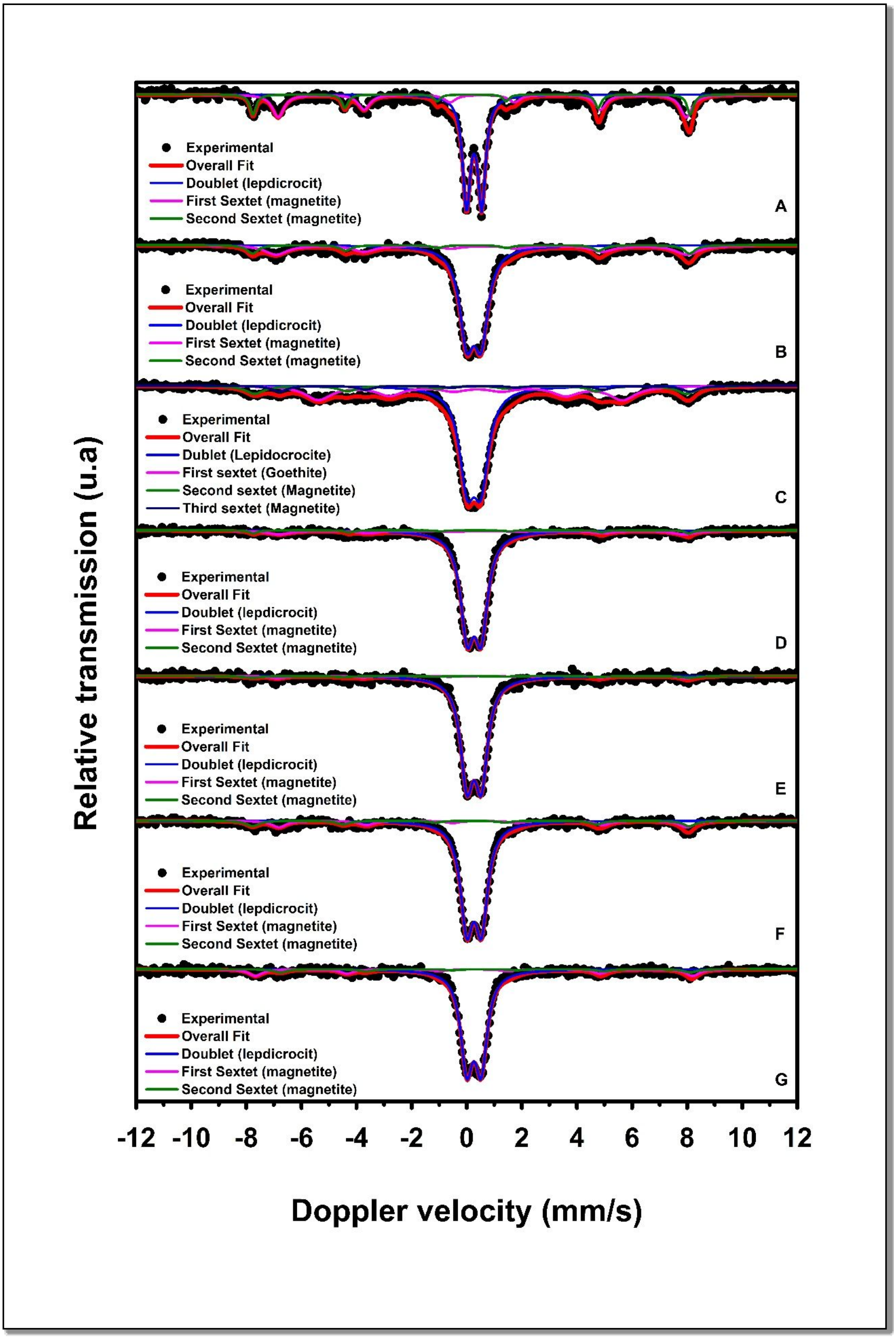

Fig. S1: Mössbauer spectra to confirm iron oxides/oxyhydroxides from immersion in saline solution without (PILs) (A), with the addition of PILs PIL 01 (B), PIL 02 (C), PIL 03 (D), PIL 04 (E), PIL 05 (F), PIL 06 (G)

Similar spectral features were observed for samples containing PILs 01, 03, 04, 05, and 06, which also exhibited a lepidocrocite-related doublet and magnetite-associated sextets. However, a relative reduction in the spectral area associated with magnetite was observed compared to the blank sample, indicating a modification in corrosion product distribution induced by the presence of PILs. In contrast, the sample containing PIL 02 exhibited an additional magnetic sextet, which can be attributed to the formation of goethite (α-FeOOH), suggesting a distinct influence of this PIL on the corrosion product evolution.

The doublet reflects the paramagnetic nature of lepidocrocite, while the magnetite sextets indicate magnetically non-equivalent Fe^{2+} and Fe^{3+} sites within its inverse spinel structure. This correlation between Mössbauer spectroscopy and XRD is essential for confirming both the composition and structural features of the corrosion products. For example, there are some phases that can be identified by X-ray diffraction, but the values are still unclear.

The Mössbauer test serves to confirm whether the identified phase indeed corresponds to that detected by X-ray diffraction (XRD). A prominent example of an iron oxide/hydroxide that can be detected by XRD but necessitates complementary techniques for precise phase identification is maghemite (γ-Fe_2O_3). Due to its crystallographic similarity to magnetite (Fe_3O_4), maghemite exhibits XRD patterns that are nearly indistinguishable from those of magnetite, rendering their differentiation by this technique alone particularly challenging.

Nevertheless, these phases differ in their iron oxidation states and magnetic properties, which can be more accurately discerned through Mössbauer spectroscopy. In such analyses, magnetite typically yields two sextets corresponding to Fe^{2+} and Fe^{3+} at distinct crystallographic sites, whereas maghemite, composed exclusively of Fe^{3+}, presents a characteristic sextet with a distinct hyperfine field [1]. With this, the understanding of the significance of using the technique becomes clear (details in Figure S1 and Table S1).

Table S1

Hyperfine parameters of the blank sample without corrosion inhibitor (0), samples 1 and 2 (PILs 01 and 02), samples 3 to 6 (PILs 03 until 06).

	d_{Fe} (mm/s)	D (mm/s)	B_{HF} (T)	G_{Fe} (mm/s)	Area (%)
Sample 0					
Dubleto	0.38	0.55	-	0.34	51.82
Sexteto 1	0.67	-0.01	45.91	0.55	32.51
Sexteto 2	0.39	-0.03	49.21	0.27	15.66
Sample 1	d (mm/s)	D (mm/s)	BHF (T)	G (mm/s)	Área (%)
Dubleto	0.37	0.51	-	0.65	51.41
Sexteto 1	0.65	-0.03	46.06	0.65	6.23
Sexteto 2	0.29	-0.08	48.53	0.77	11.20
Sexteto 3	0.37	-0.29	34.09	1.37	31.60
Sample 2	d (mm/s)	D (mm/s)	BHF (T)	G (mm/s)	Área (%)
Dubleto	0.37	0.52	-	0.62	66.20
Sexteto 1	0.68	-0.06	46.22	1.01	23.56
Sexteto 2	0.29	-0.04	49.18	0.49	10.24
Sexteto 3	0.35	-0.28	32.09	1.30	30.55
Sample 3	d (mm/s)	D (mm/s)	BHF (T)	G (mm/s)	Área (%)
Dubleto	0.38	0.51	-	0.57	83.87
Sexteto 1	0.71	-0.013	45.62	1.07	13.05
Sexteto 2	0.35	-0.015	49.02	0.32	3.08
Sample 4	-	-	-	-	-
Dubleto	0.37	0.52	-	0.53	80.44
Sexteto 1	0.67	-0.02	46.1	0.59	13.24
Sexteto 2	0.26	-0.02	49.2	0.59	6.32
Sample 5	-	-	-	-	-
Dubleto	0.38	0.52	-	0.55	90.37
Sexteto 1	0.67	-0.02	46.1	0.65	5.16
Sexteto 2	0.26	-0.02	49.2	0.77	4.47
Sample 6	-	-	-	-	-
Dubleto	0.37	0.53	-	0.55	78.60
Sexteto 1	0.73	-0.06	45.91	0.65	12.42
Sexteto 2	0.24	0.04	49.15	0.56	8.98

The combined application of XRD and Mössbauer spectroscopy is essential for the reliable identification of these phases, thereby ensuring the accurate characterization of corrosion products. While Mössbauer spectroscopy provides detailed insights into the oxidation states, magnetic interactions, and local environments of iron species, XRD complements this by identifying and confirming the crystalline phases and their lattice parameters. In the present case, the identification of lepidocrocite by Mössbauer spectroscopy is consistent with its characteristic diffraction peaks observed in the XRD patterns, thereby confirming its presence. Similarly, the detection of magnetite by both techniques reinforces the reliability of the results, as the hyperfine interactions identified through Mössbauer spectroscopy align with the cubic inverse spinel structure revealed by XRD. This integrative approach ensures a robust and accurate characterization of the corrosion products formed in the blank sample.

Further Mössbauer analysis of samples 1 and 2, treated with PILs 01 and 02, respectively, revealed spectra composed of two main components: one doublet and three sextets (details in Figure S1). Based on the hyperfine parameters and supported by X-ray diffraction (XRD) data, the doublet was attributed to lepidocrocite, two sextets to magnetite, and the remaining sextet to the goethite phase. These findings, together with the previous analyses, confirm the presence of goethite among the corrosion products formed in these systems. The following sections discuss chloride permeability beneath the carbon steel surface in light of these observations.

In contrast, samples 3, 4, 5, and 6 exhibited Mössbauer spectra composed of one doublet and two sextets, similar to those observed for the blank sample; however, the sextets presented lower relative areas. Based on the hyperfine parameters and XRD results, the doublet was assigned to lepidocrocite, while the sextets correspond to magnetite (Fig. S1 and Table S1), corroborating the phase identification obtained by XRD. The reduced magnetite contribution suggests differences in the formation and distribution of corrosion products in these systems. The interpretation of the Mössbauer spectra was guided by the hyperfine parameters reported by Murad (Table S2), which were used as reference values for phase identification.

Table S2
Reference hyperfine parameters (adapted from Murad, Mössbauer Spectroscopy of Environmental Materials and Their Industrial Utilization).

	d (mm/s)	D (mm/s)	BHF (T)
Lepdocrocita	0.37	0.53	-
Magnetita 1	0.26	≤ |0.02|	46.1
Magnetita 2	0.67	≤ |0.02|	49.2
Goetita	0.37	-0.26	38

In sum, still about table 16, in the literature, these parameters are considered fundamental for distinguishing among the various oxidation states and coordination environments of iron. Notably, the contributions of Murad are particularly significant in providing the reference values required to accurately identify specific phases such as magnetite, lepidocrocite, and goethite; based on Mössbauer spectroscopy data.

2 Assessment of the rust layer

2.1 Macroscopic technique for oxide/oxyhydroxide layer evaluation

The application of optical microscopy techniques is recognized as a fundamental source for the evaluation of corrosion products formed on carbon steel surfaces. This methodology offers advantages that make it particularly suitable for the preliminary characterization of CP in research and industrial contexts. One of the primary benefits of optical microscopy lies in its cost-effectiveness.

Compared to more complex and resource-intensive techniques, OM requires relatively simple instrumentation and low operational costs, making it accessible for routine analyses. It provides a direct and immediate visual assessment of the corroded surface, enabling researchers to conduct a preliminary evaluation of the general morphology and spatial distribution [2,3].

In addition to its practicality, optical microscopy facilitates the identification of important visual characteristics of the corrosion layer, such as variations in texture, color, and the overall pattern of oxide formation. These features can yield valuable insights into the underlying corrosion mechanisms responsible for the observed degradation.

For instance, homogeneity or heterogeneity in color and texture can suggest localized corrosion, pitting, or uniform corrosion processes. Similarly, certain morphological patterns may be indicative of specific oxide phases, aiding in the initial hypotheses about the composition and structure of the corrosion products. These visual clues, although not definitive on their own, can be crucial for guiding subsequent, more detailed analyses [2,3].

Another significant advantage of using optical microscopy is its ability to reveal the stratification of oxide/oxyhydroxide layers. This technique enables the visual distinction of corrosion-induced phases, facilitating assessment of their protective efficacy. Uniform, continuous oxide layers may indicate effective barrier formation, while porous or discontinuous layers suggest limited protection. Thus, optical microscopy provides a crucial preliminary evaluation of the corrosion product layer’s integrity and performance.

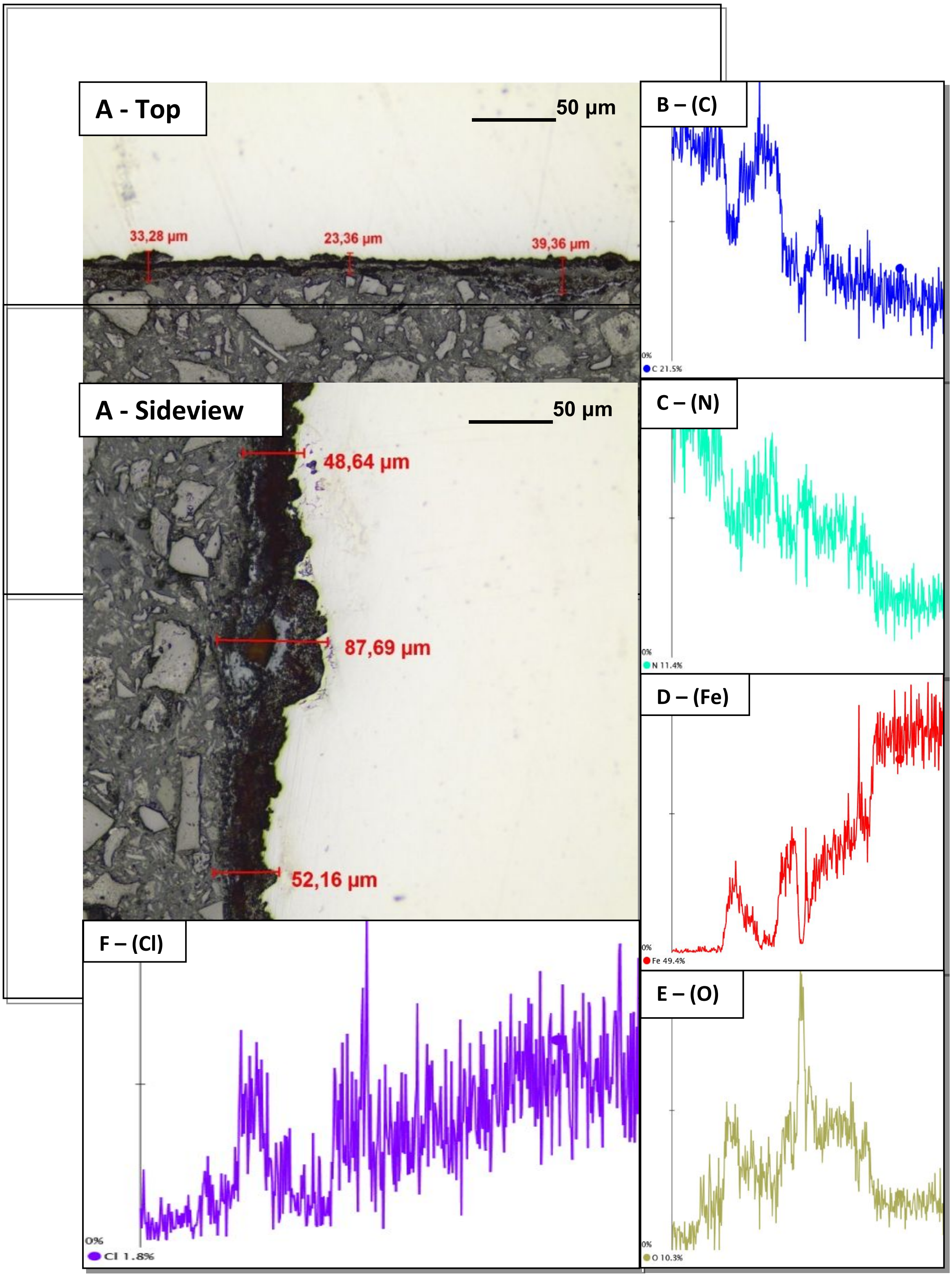

Fig. S2. Evaluation of the corrosion product thickness. (A) Optical microscopy images showing side and top views used to assess the layer's thickness without corrosion inhibitors addition (PILs). (B–F) Elemental analysis performed by Energy Dispersive Spectroscopy (EDS) using line scan mode to determine the distribution of key elements across the corrosion layer.

In Figure S2, particularly in region A, the average thickness of the oxide/oxyhydroxide layer is observed to be less than 50 μm, indicating a relatively thin formation. A significant advantage of optical microscopy is its non-destructive nature, which enables the examination of samples without compromising their structural or chemical integrity. This characteristic allows the same specimens to be subsequently analyzed using higher-resolution techniques such as scanning electron microscopy (SEM) and energy-dispersive X-ray spectroscopy (EDS), thereby facilitating a comprehensive, multi-technique characterization of the corrosion products.

In the present study, optical microscopy was employed to investigate the corrosion products formed on the surface of carbon steel. Specifically, the oxide layer on the reference (blank) sample was examined, as illustrated in Figure S2. The micrograph provided clear evidence of oxide formation, and the thickness of this layer was quantitatively estimated using SmartVisio software, allowing for a more precise evaluation of oxide growth [4]. To detail the experimental procedure, carbon steel specimens were exposed to a 3.5% NaCl solution, both with and without the addition of corrosion inhibitors (protic ionic liquids, PILs). Following exposure, the samples were embedded for cross-sectional analysis to determine the thickness of the oxide/oxyhydroxide layers formed. Additional objectives included assessing potential surface degradation, quantifying the oxide layer thickness, and determining whether visible distinctions could be made among the oxide products, based on previously established phase identification under the tested conditions.

As shown in Figure S2, the analyzed sample contains two distinct regions, labeled A and B. Region A corresponds to the upper portion of the sample, while region B represents the lateral area. Identifying the specific region under analysis is essential for interpreting the subsequent effects of corrosion inhibitors, as discussed in Figures S3 and 40. In the absence of corrosion inhibitors, only limited oxyhydroxide phases primarily lepidocrocite and magnetite were detected, which corresponded to relatively low layer thicknesses. Differences in corrosion product coloration were also observed. EDS analysis was conducted to determine elemental composition within the corrosion layer. In region A, severe degradation reaching approximately 420 μm was observed, attributed to chloride ion penetration, which accelerated the corrosion process. Region B exhibited similar surface irregularities, including rounded areas near the sample’s edge, further evidencing the aggressive influence of excess chloride ions in the electrolyte.

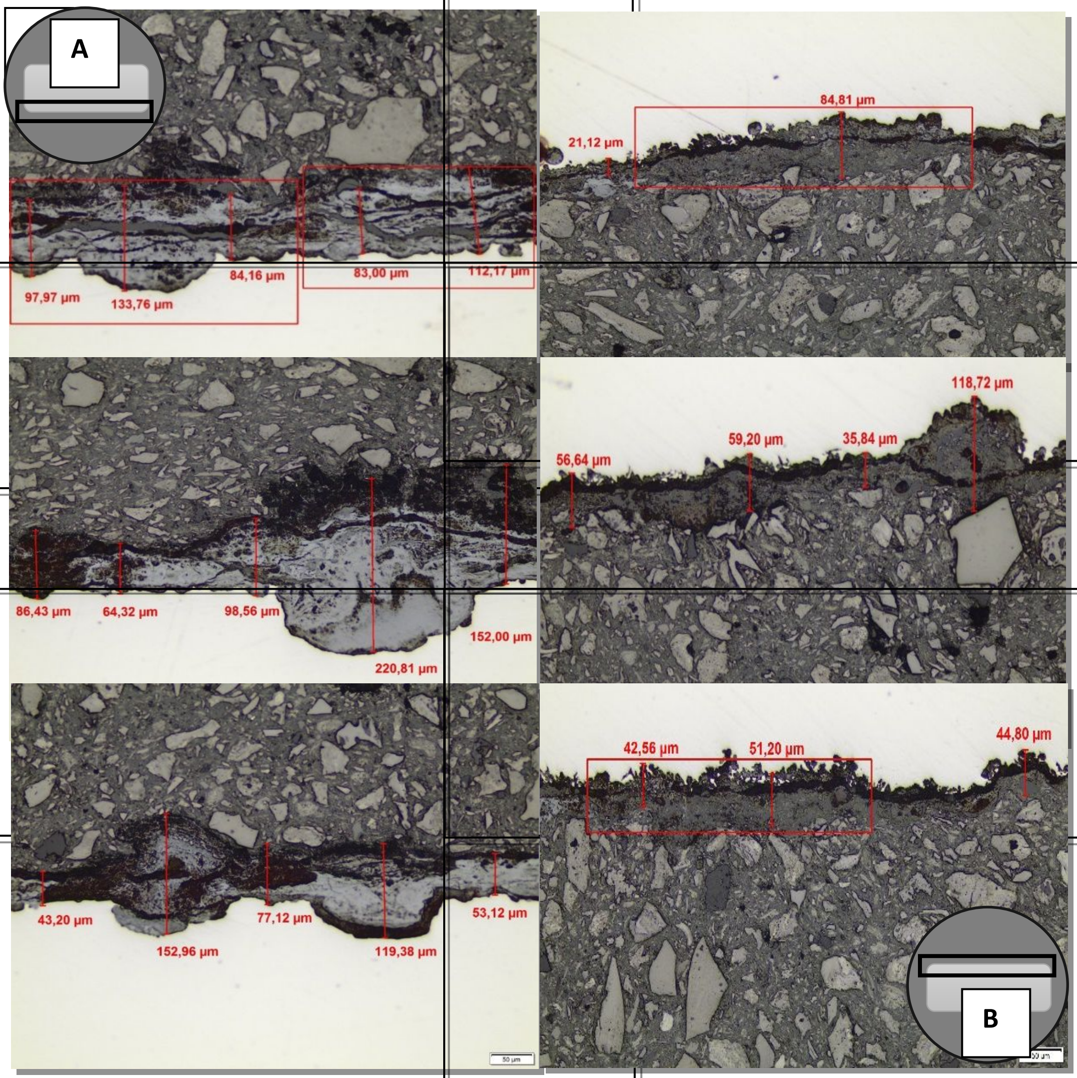

Fig. S3 - Evaluation of the corrosion product thickness. (A and B) Optical microscopy images showing side and top views used to assess the layer's thickness without corrosion inhibitors addition (PILs).

Figure S3 presents the analysis of oxides and oxyhydroxides through optical microscopy. The addition of the protic ionic liquid, specifically PIL 01, resulted in the development of a thicker layer of corrosion products. This increased layer thickness is attributed to the formation of goethite alongside lepidocrocite and magnetite, which occurs in the presence of the corrosion inhibitor.

In neutral to slightly alkaline environments (i.e., at higher pH values), the formation of goethite (α-FeOOH) is often thermodynamically favored over other iron oxyhydroxides such as lepidocrocite, while phases such as hematite may form under different environmental conditions. Under highly alkaline conditions, the formation of additional mineral phases, including magnetite (Fe_3O_4), may also occur. Furthermore, increasing temperature can promote the nucleation and growth of goethite, with accelerated formation and the development of larger crystals typically reported at temperatures above approximately 70–80 °C [5,6].

At elevated temperatures, the formation of hematite (Fe_2O_3) is generally thermodynamically favored due to its lower Gibbs free energy under such conditions, and it is commonly considered a stable end-product of high-temperature oxidation in iron-based systems. Nevertheless, in environments characterized by slightly alkaline pH and moderate temperatures, goethite frequently appears as one of the dominant corrosion products. Under these conditions, the stability and persistence of this phase are associated with the combined influence of thermodynamic and kinetic factors governing iron oxyhydroxide formation.

The preferential formation of specific iron oxide or oxyhydroxide phases is therefore strongly influenced by environmental parameters, particularly pH and temperature, which not only determine the thermodynamic feasibility of these phases but also control the kinetics of nucleation and growth. Consequently, even relatively small variations in these parameters may significantly affect the sequence and distribution of corrosion products. Localized changes in pH caused by electrochemical reactions, slight variations in temperature, or differences in dissolved oxygen availability may promote the coexistence or preferential development of different iron-containing phases, as illustrated in Figure S3.

Among these phases, lepidocrocite (γ-FeOOH), magnetite (Fe_3O_4), and hematite may develop as secondary or competing corrosion products, contingent upon the specific electrochemical and physicochemical conditions prevailing at the metal–solution interface. The corrosion products in Section A of the examined sample are arranged in stratified layers that exhibit considerable variation in both morphology and thickness. Quantitative analysis indicates that the average thickness of the corrosion product layer is approximately 80 μm. Although this layered structure appears constant in certain areas, significant heterogeneities are evident through visual observation and microscopic evaluation. Specifically, variations in coloration across the oxide/oxyhydroxide layer indicate differences in either the chemical composition or the crystallographic nature of the corrosion phases present. These chromatic discrepancies may indicate localized electrochemical variations during the exposure period, potentially resulting from uneven distribution of corrosion currents, pH gradients, or differential oxygen diffusion at the metal surface. Furthermore, such heterogeneity may be attributed to intrinsic microstructural characteristics of the substrate such as grain boundaries, inclusions, or phase segregation that influence the nucleation and growth dynamics of the corrosion products [7–9].

The heterogeneous morphology and structure of the corrosion layers highlight the intricate interaction between the microstructural characteristics of the material and the surrounding environmental conditions. This interplay is ultimately responsible for the morphology, thickness, and chemical composition of the corrosion products that develop. Although Figure S4 depicts extensive degradation of the exposed carbon steel surface in this region, the introduction of corrosion inhibitors has evidently facilitated the formation of a denser and more compact corrosion product layer in the affected area. The observed compactness and visual uniformity of this layer suggest the partial establishment of a protective barrier, likely attributable to the inhibitors' capacity to promote the development of more stable and adherent oxide phases.

Despite persistent variations in the thickness and composition of the oxide layer across the surface, regions exhibiting mechanical rupture or detachment frequently expose a darker underlying layer covering the substrate. This darker tone is indicative of magnetite (Fe_3O_4) formation, consistent with findings reported in the literature [10,11]. The localized presence of magnetite in these areas may contribute to the structural consolidation of corrosion products, resulting in a more cohesive and integrated corrosion layer.

In contrast, Section B demonstrates more pronounced and widespread surface degradation, which appears to correlate with the absence or diminished thickness of protective oxide layers. The average thickness of the corrosion layer in this region was measured to be slightly lower, approximately 60 μm, particularly in the lower portion of the sample. This reduction in oxide/oxyhydroxide layer formation may be associated with the sample's positioning during immersion, potentially leading to suboptimal distribution or limited adsorption of the inhibitor molecules in the peripheral zones of the carbon steel surface.

Additionally, a marked difference in color is observed in Section B, where the corrosion products exhibit a significantly darker tone relative to the adjacent Bakelite. This may again suggest localized formation of magnetite or other dense oxide phases, further underscoring the uneven distribution of protection across the material surface. In fact, Figure S4 presents the analysis of corrosion products conducted primarily through optical microscopy. Accordingly, the incorporation of protic ionic liquid PIL 02 into the saline electrolyte resulted in the formation of a distinct corrosion product layer.

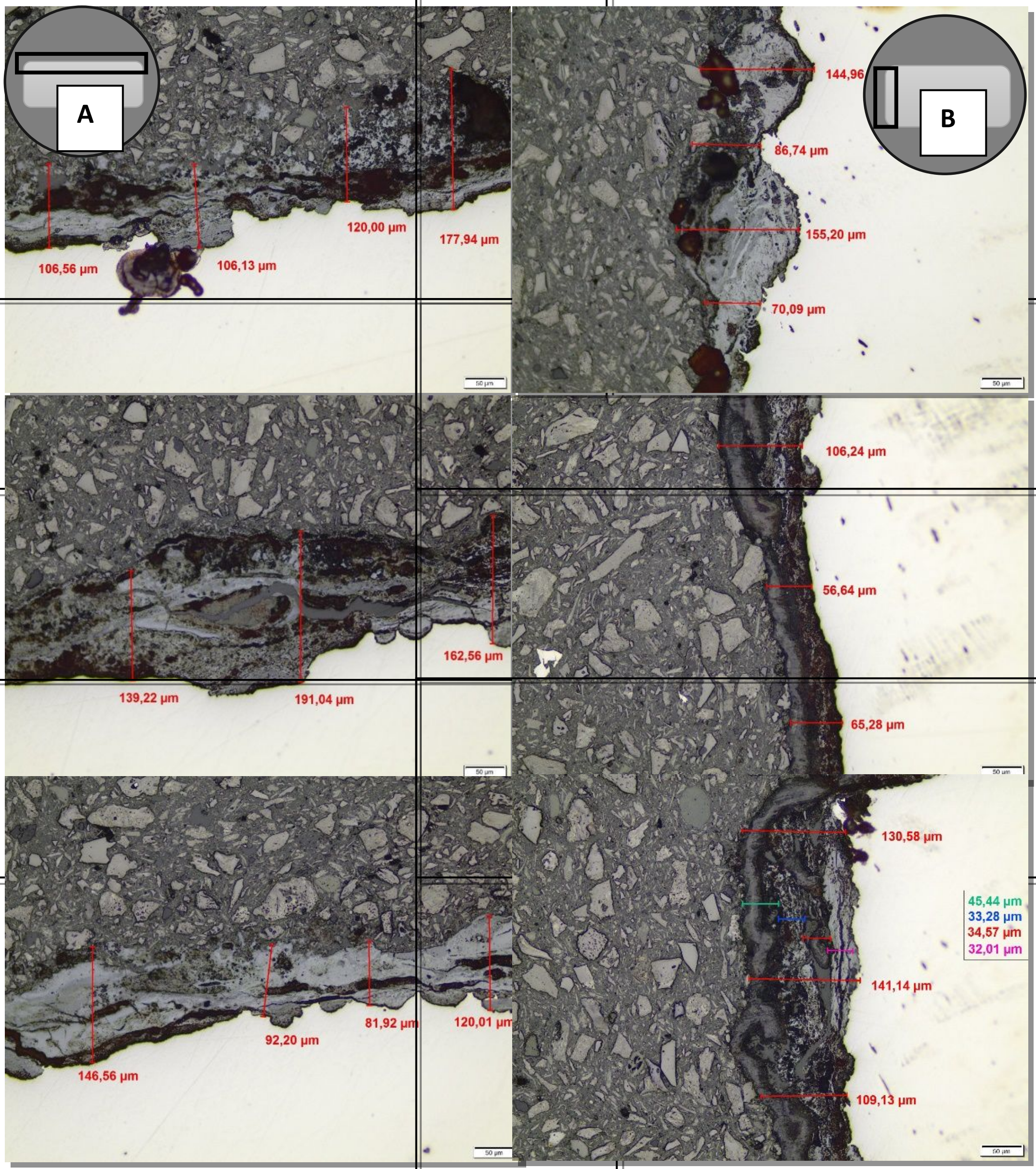

Fig. S4 - Evaluation of the corrosion product thickness. (A and B) Optical microscopy images showing side and top views used to assess the layer's thickness with corrosion inhibitors addition (PIL01 - HEAF).

The formation of this compact and thicker oxyhydroxide layer is associated with the presence of corrosion inhibitors, which promotes the development of a specific iron oxyhydroxide phase goethite alongside lepidocrocite and magnetite. As shown in Figure S3, section A, corrosion products of varying sizes can be observed, with the greatest average layer thickness, measuring approximately 100 μm, recorded in the immersion experiments.

The presence of distinct color variations across the oxide/oxyhydroxide layers clearly indicates that compositional or structural heterogeneity within the corrosion products. These visual distinctions are particularly important, as they indicate changes in the corrosion mechanisms and the potential protective behavior of the formed layers. Despite the pronounced surface degradation depicted in Figure S3, which illustrates the extent of corrosive damage under uninhibited conditions, the incorporation of corrosion inhibitors significantly improves the material's condition. Notably, the application of the Protic Ionic Liquid (PIL 02), as presented in Figure S5, results in a substantial transformation: a dense and compact layer develops in the affected region, suggesting an enhanced capacity for corrosion resistance. The absence of a thicker oxide/oxyhydroxide layer is associated with more aggressive and widespread material degradation. The average thickness in this section is significantly lower than in others, measuring approximately 60 nm, particularly in the lower portion of the sample. This limited oxide formation may be attributed to the sample's positioning during immersion, which could have impacted on the uniform distribution and adherence of inhibitor molecules. Furthermore, regions with reduced exposure or less favorable surface interactions exhibited diminished corrosion protection. Supporting this interpretation is the pronounced color contrast observed in Section B, where the corrosion product appears significantly darker than the surrounding bakelite, potentially indicating the presence of distinct corrosion phases or variations in oxide density.

In Figure S5, further quantitative evaluation of these observations is provided. By utilizing optical microscopy, the thickness of the various corrosion product layers on the carbon steel surface was estimated. For systematic evaluation, Figure S5 was segmented into three regions, designated A through C. Sections A and B correspond to areas exposed to PIL 01, where the total thickness of the corrosion product layer was approximately 70 µm. Throughout this layer, distinct internal sublayers were identified. Accordingly, to accurately determine their dimensions, markings were employed to delineate and measure these intermediate sections. The analysis indicated that the average thickness of these sublayers 25 µm. These findings are critical, as they suggest that the formation of multi-layered oxides/oxyhydroxides contributes to the development of a more compact and potentially protective surface film. Nevertheless, such multilayered structures were absent in samples subjected to 24-hour immersion without inhibitors. This preliminary optical microscopy assessment provides a foundational basis for further investigations using more advanced characterization methods.

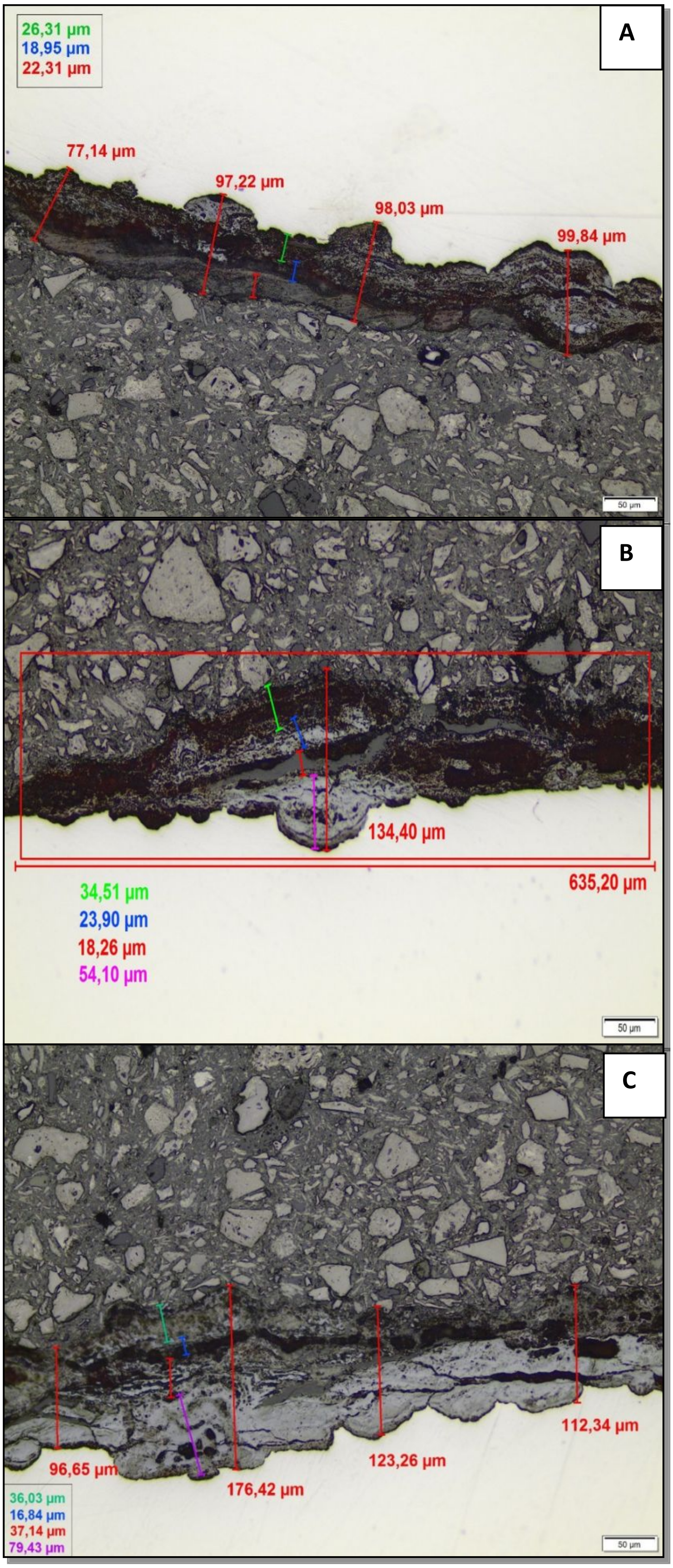

Fig. S5 - Evaluation of the corrosion product thickness. (A and C) Optical microscopy images showing side and top views used to assess the layer's thickness with corrosion inhibitors addition (PIL02 - HDEAF).

7.2.2. Elemental analysis by line spectra of rust section from microscopic techniques

The Energy Dispersive Spectroscopy (EDS) technique, particularly when applied through line scan analysis, plays a pivotal role in corrosion assessment due to its capability to determine the elemental composition along a defined line or region across the sample surface. This level of precision is especially important in corrosion studies, where the formation of distinct phases necessitates a detailed understanding of their elemental makeup. In this context, the detection of specific elements such as oxygen, chlorine, or sulfur alongside metals enables EDS line scans to identify the nature of corrosion products, including oxides, oxyhydroxides, chlorides, or sulfides. This information is essential for elucidating the underlying mechanisms driving the corrosion process [12,13].

EDS line scan analysis is a valuable tool for characterizing the longitudinal distribution of elements across corroded surfaces or within distinct layers of corrosion products. This spatially resolved data facilitates the identification of corrosion morphologies such as pitting, crevice corrosion, or uniform attack, while also providing critical insights into the interface between the metallic substrate and the overlying corrosion layers. Examination of this interfacial region is essential for assessing the protective or deleterious nature of the formed products, enabling correlations between elemental composition and microstructural features such as grain boundaries or inclusions that may influence corrosion behavior. In addition to its qualitative capabilities, EDS line scan yields semi-quantitative information, allowing for the estimation of elemental concentrations across various regions and aiding in the evaluation of corrosion extent or compositional gradients within the corrosion products.

In Figure S6, distinct elemental behaviors were observed along the sample during the EDS line scan analysis conducted on the blank specimen, which did not contain CI in the solution. This analysis provides a visual analysis of the formation of corrosion residues at the interface between the metallic substrate and the Bakelite mounting material. In this context, the concentration of carbon (C) initially exhibited elevated values. As the scan progressed, a gradual decrease in carbon content was observed, which is attributed to the formation of corrosion products.

These products are relatively dense and impede the diffusion of carbon, leading to a subsequent stabilization of its concentration toward the end of the scan. In contrast, iron (Fe) displayed a more complex profile, characterized by an initial increase in concentration, followed by a decrease, and then a subsequent rise toward the end of the measurement.The prime conclusion from this evaluation is the presence of distinct spacings and morphologies of CP, each characterized by varying iron contents. These compositional differences are reflected in the quantification results obtained through the EDS line-scan technique, particularly as the scan progresses toward the interior of the substrate. Among the key elements analyzed in this section, chlorine (Cl) exhibited a notable increase in concentration along the scanned region. This observation suggests that the corrosion layer formed in this environment exhibits relatively high permeability to chloride ions. Such behavior may be attributed to the nature of the corrosion products identified in this region, particularly the presence of magnetite and lepidocrocite. According to existing literature, elevated levels of lepidocrocite are associated with increased chloride permeability due to its more open and loosely packed structure compared to other corrosion products [14].

Oxygen (O) showed an initial rise in concentration, followed by a decline and subsequent stabilization. This trend is likely associated with the formation of iron oxides and oxyhydroxides, as the oxygen content in these compounds varies depending on their specific stoichiometry and distribution across the external region of the specimen. Nitrogen (N) exhibited a continuous decrease in concentration throughout the scanned profile. It is important to highlight that this behavior differs significantly from that observed in Figure S6, where the EDS line-scan analysis of the corrosion product in the presence of a corrosion inhibitor revealed an increasing nitrogen signal, attributed to the presence of nitrogen-containing (PILs) employed as corrosion protection agents for carbon steel.

These variations in elemental concentrations across the analyzed region provide important insights into the chemical interactions and processes occurring at the corroded metal–solution interface, thus contributing to a better understanding of the corrosion mechanisms involved. The application of EDS is particularly valuable in this context, as it enables both qualitative and quantitative assessment of the elements present on the corroded surface. This analytical capability is essential for elucidating the composition of corrosion products and assessing the extent of material degradation.

In immersion conditions involving NaCl, the evaluation of specific elements namely Fe, Cl, O, C, N, and Na is critical, given their direct relevance to corrosion phenomena. Each of these elements plays a fundamental role in interpreting corrosion behavior, and accurate identification of their spatial distribution is vital for a thorough and meaningful discussion of the results.

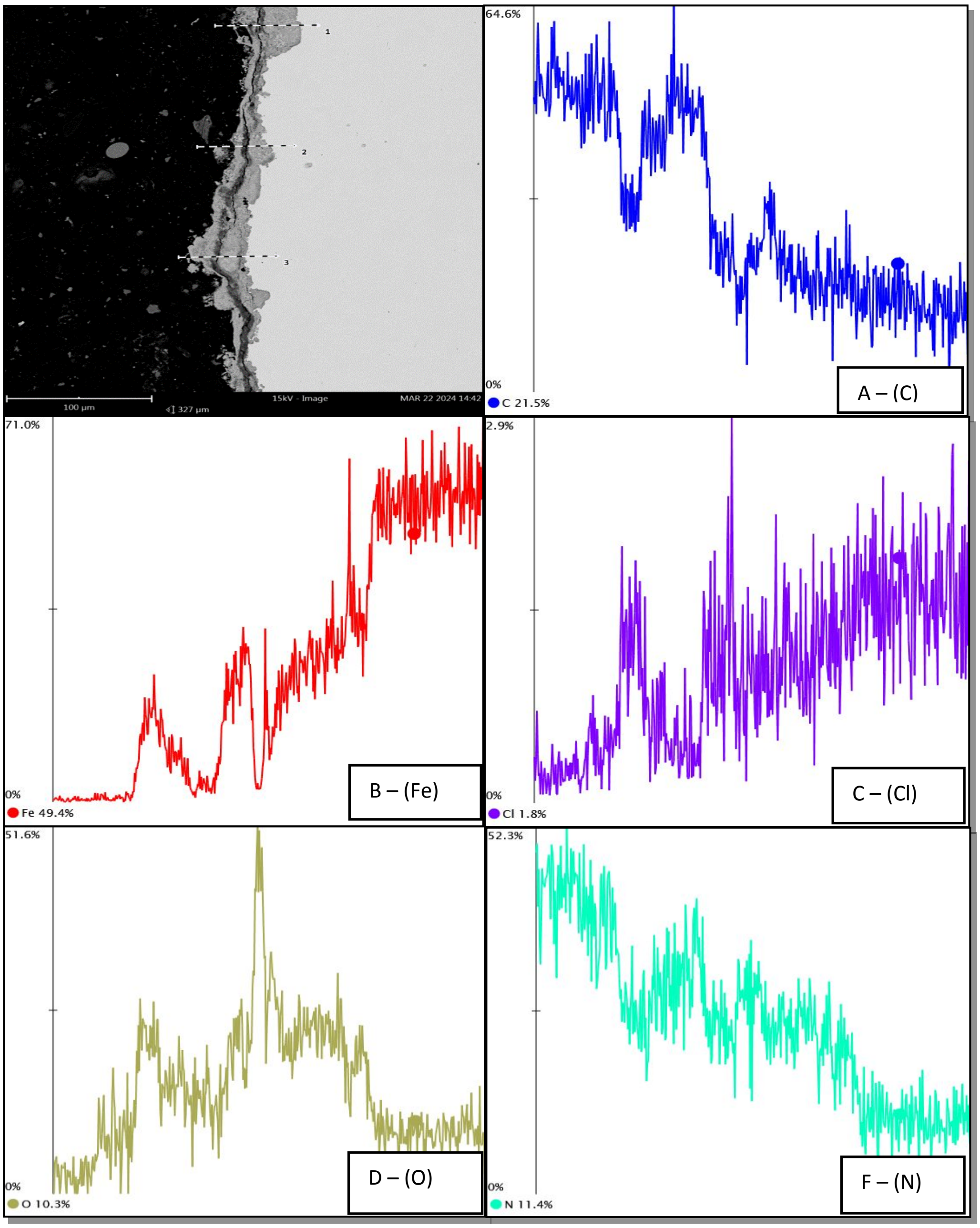

Fig. S6 - Energy-Dispersive X-ray Spectroscopy (EDS) line-scan analysis of the carbon steel surface after 24 h of immersion in a 3.5 wt.% NaCl solution in the absence of protic ionic liquids (Blank sample). SEM cross-sectional image indicating the region selected for the line-scan analysis. Corresponding elemental intensity profiles for (A) C , (B) Fe, (C) Cl, (D) O, and (F) N, showing the variation in elemental distribution across the corrosion layer and substrate interface.

Iron (Fe), the principal constituent of carbon steel, plays a fundamental role in identifying either its metallic state or oxidized forms, such as iron oxides, which are essential for assessing the extent of corrosion. The detection and spatial distribution of oxygen (O) serve as critical indicators of the formation and progression of iron oxides or hydroxides within corrosive environments.

These compounds may be associated with the development of passive protective films or, alternatively, with the generation of less stable corrosion products, depending on the surrounding environmental conditions and the electrochemical interactions occurring at the metal–electrolyte interface. The spatial distribution of oxygen provides essential insights into both the nature and extent of corrosion, as well as the potential development of protective barrier layers that may inhibit further material degradation. Chlorine (Cl), by contrast, is widely acknowledged as one of the most aggressive corrosive agents affecting steel. Its presence within corrosion products often signifies the formation of soluble and highly reactive compounds, such as iron chlorides.

These species are known to actively contribute to localized corrosion phenomena, including pitting, thereby further undermining the structural integrity of the metal. Consequently, the detection of chlorine in corrosion products is critical for evaluating the severity and propagation of corrosion processes. Carbon (C) serves multiple roles in corrosion studies. It can act as an indicator of organic materials or carbonaceous residues that may originate from external contamination or from corrosion inhibitors. Additionally, the identification of carbonates formed during corrosion reactions through carbon signals can offer further evidence of specific degradation mechanisms.

Although less frequently detected, nitrogen (N) may be present in corrosion environments contaminated by nitrogen-containing substances, such as ammonia or organic nitrogen compounds. Its detection may point to specific corrosion pathways associated with nitrogenous species. In the context of the Energy Dispersive Spectroscopy (EDS) line scan analysis presented in Figure S6, which corresponds to tests conducted with the addition of corrosion inhibitors, each element exhibited a distinct distribution pattern.

Initially, carbon (C) appeared in high concentrations, attributed to the presence of Bakelite. As the scan progressed and corrosion products began to develop, a marked decline in carbon intensity was observed, indicating that these products hindered the diffusion of carbon from the resin. Following this reduction, the carbon signal stabilized with only minor fluctuations. The behavior of iron (Fe) was more complex. An initial increase in its concentration was followed by a stabilization period, a subsequent decline, and a final slight increase near the end of the scan. This pattern is likely associated with variations in the thickness and composition of the oxide and oxyhydroxide layers, which differ in iron content.

Notably, in the presence of the most effective inhibitor (PIL A), the formation of goethite a more stable iron oxyhydroxide was observed. This phase typically forms under controlled pH and temperature conditions, aligning with one of the study's primary objectives: to investigate the formation of denser corrosion deposits composed of more stable corrosion products on the steel surface (as detailed in Figure S7).

One of the primary elements assessed in this section was chlorine (Cl). Upon the addition of corrosion inhibitors, a noticeable decline in chlorine concentration was observed, followed by stabilization, indicating a more consistent and uniform formation of corrosion products over time. Although the overall chlorine levels were not elevated, they were notably reduced in comparison to the uninhibited system.This behavior suggests that, in the presence of the corrosion inhibitor (PIL A), the development and accumulation of the corrosion layer is significantly diminished relative to conditions with uninhibited chloride exposure. This mitigating effect may be attributed to the formation of specific oxyhydroxide phases, such as goethite, which were identified through differential microscopy techniques applied in this region.

The observed reduction in chlorine presence highlights the inhibitor's influence in altering the local electrochemical environment, thereby reducing the availability and retention of chloride ions on the surface of the metal, which would otherwise promote active corrosion processes. According to the literature, elevated levels of this type of corrosion product are commonly linked to the development of a more compact and uniform protective layer.

This densification contributes to the formation of a more effective barrier against the penetration of aggressive species such as chloride ions. The presence of such compact oxide or oxyhydroxide phases, especially those formed under inhibited conditions, may limit the diffusion pathways for ionic species and hinder the propagation of localized corrosion. As the corrosion products accumulate, their structural and morphological characteristics directly influence the degree of protection afforded to the underlying metal. A more compact layer not only minimizes porosity but also enhances adhesion to the metal substrate, thereby improving the long-term performance of the protective barrier. This compact effect, therefore, plays a critical role in reducing the susceptibility of the material to further corrosion, particularly in aggressive saline environments. Oxygen (O) content initially increased, followed by fluctuations and a subsequent decline, ultimately reaching a stable value toward the end of the analysis. This behavior can be attributed to the sequential formation and transformation of various iron oxides and oxyhydroxides during the corrosion process.The observed variations in oxygen concentration reflect the dynamic evolution of these corrosion products, which differ in stoichiometry and structural complexity. The presence and distribution of oxygen are indicative of the nature and extent of oxidation occurring on the metal surface, as well as the relative stability of the phases formed.This variation is particularly significant in understanding the kinetics of oxide layer development and the inhibitor's influence on phase composition. The oxygen profiles serve as an important marker for identifying the depth and density of oxide films formed throughout the exposure period, providing insight into how the protective layer evolves under the influence of the corrosion inhibitor.

Nitrogen (N) exhibited an initial decrease in concentration, followed by a period of stabilization and a slight subsequent increase. The reduction is consistent with the transition toward the metallic substrate, where nitrogen is virtually absent due to the composition of the carbon steel alloy, which does not contain nitrogen as a principal element. As the EDS line scan progresses through the corrosion layer and into the metal substrate, this absence becomes more pronounced. However, in the regions associated with the protective film formed in the presence of the inhibitor, the slight reappearance of nitrogen can be attributed to the chemical composition of the inhibitor molecules, which may contain nitrogen functional groups.

These residues become integrated into the corrosion product matrix during the inhibition process. Although the quantities detected are minimal, they provide indirect

evidence of the inhibitor's incorporation into the surface layer, thereby confirming its participation in forming a barrier that modifies the chemistry and structure of the corrosion products, as validated through EDS measurements.

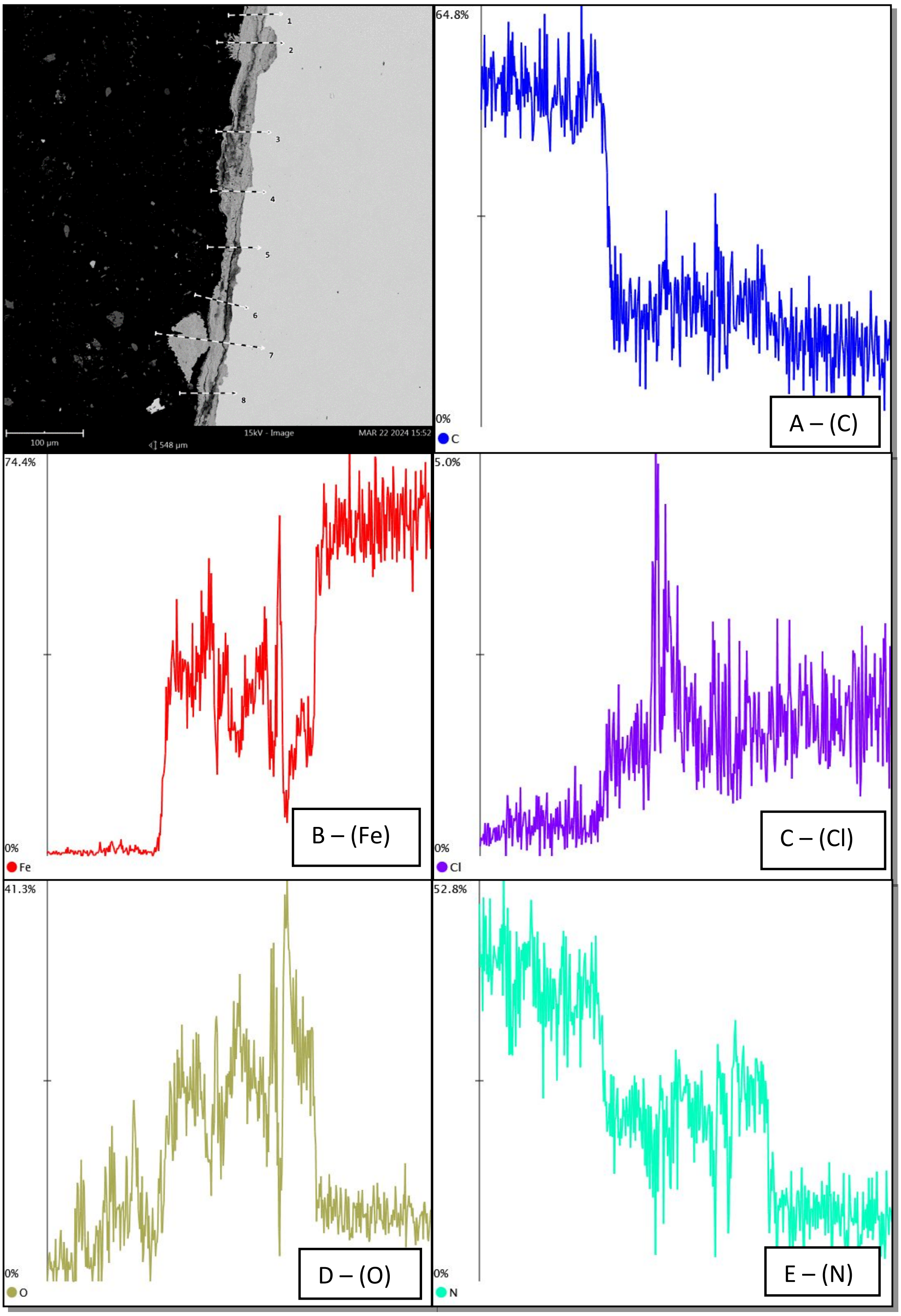

Fig. S7. Energy-Dispersive X-ray Spectroscopy (EDS) line-scan analysis of the carbon steel surface after 24 h of immersion in a 3.5 wt.% NaCl solution containing the protic ionic liquid PIL 01. SEM cross-sectional image indicating the region selected for the line-scan analysis. Corresponding elemental intensity profiles for (A) C , (B) Fe, (C) Cl, (D) O, and (F) N, showing the elemental distribution across the corrosion layer and the steel substrate interface in the presence of the corrosion inhibitor.

The literature presents several studies that employ the EDS technique as a primary method to establish a foundational understanding of the decomposition processes affecting steel when exposed to various corrosive agents, such as chloride and sulfide. These studies further explore the formation and propagation of corrosion products of the material with long periods of immersion [15–17].

The Energy Dispersive Spectroscopy (EDS) technique is essential to evaluate corrosion products, as it allows precise analysis of the elemental composition of corroded materials, identifying and quantifying critical elements to understand corrosion mechanisms. In addition to mapping the spatial distribution of the elements, EDS helps identify corrosion patterns and investigate chemical interactions in the metal-corrosion interface. Being a non -non-destructive technique, it preserves the integrity of the samples and, when combined with Scanning Electronic Microscopy (SEM), offers a more complete view of corrosive processes [18].

Besides, in literature, it is possible to find works that used the eds technique in parallel with the results of optical microscopy and scanning electronics in the study of corrosion inhibitors. Thus, this assessment is crucial to the material field considering that the estimated quantification of elements can provide even the elements present in selected regions with these results, a more concise explanation of details is possible to reinforce the study of the mechanism of inhibition [19,20].

Thus, extending the discussion about EDS line scan analysis, the behavior of each element varied significantly across the sample. In this context, the evaluation of the carbon element (C) began with high concentration values, and as the evaluation progressed there was a reduction due to the formation of the oxide/oxyhydroxide layer, not allowing the passage of carbon from Bakelite (details in figure S7).

Furthermore, iron (Fe) exhibited a more intricate behavior, characterized by an initial increase in concentration, followed by fluctuations, a subsequent decrease, and a slight increase towards the end of the scan. In contrast to what was observed in PIL 02 (B), this behavior can be recognized as the varying spacing and the formation of oxides and oxyhydroxides, which contain different iron concentrations in their composition. Notably, with the addition of the corrosion inhibitors, a new form of oxyhydroxide (goethite) was detected.

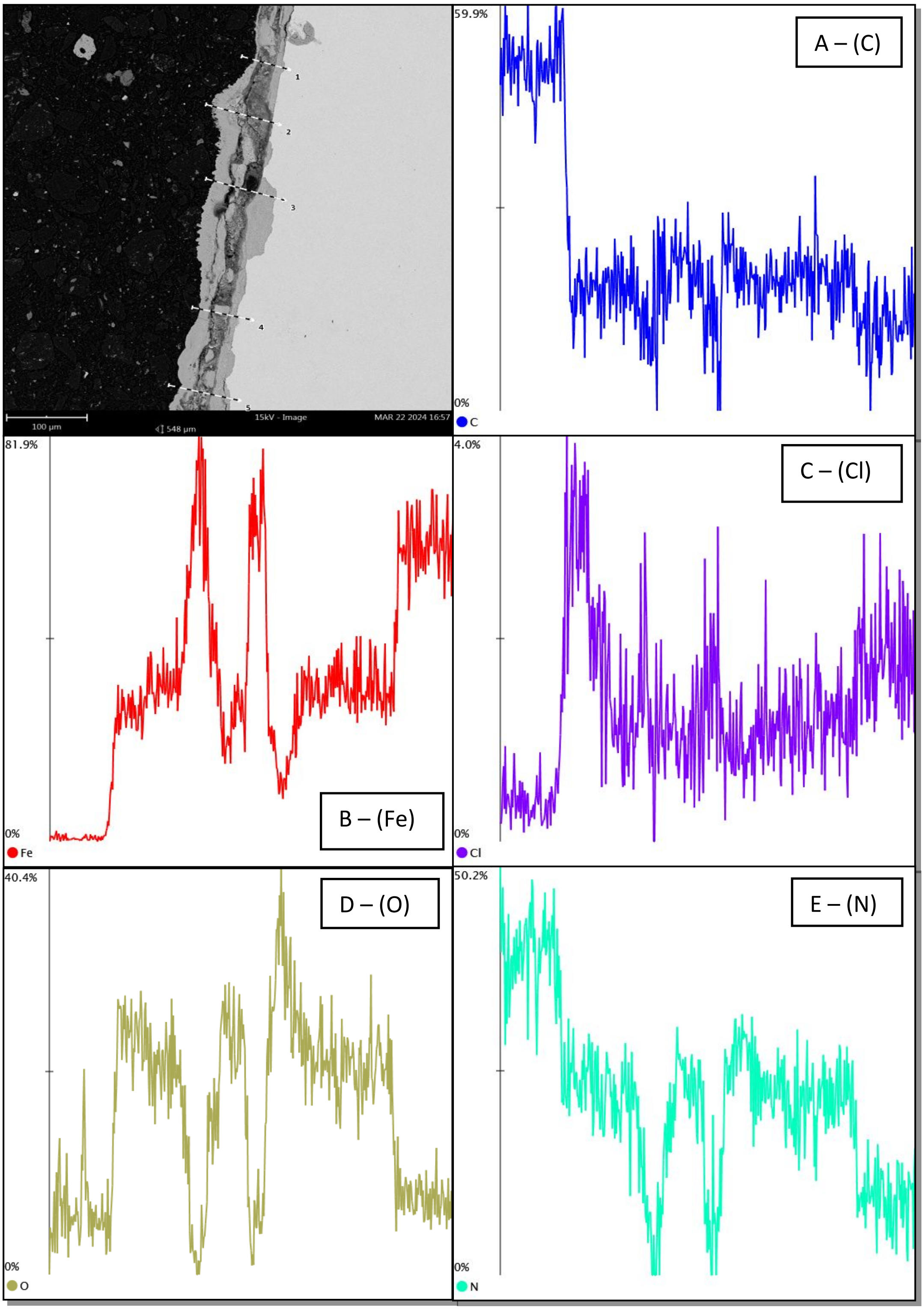

Fig. S8. Energy-Dispersive X-ray Spectroscopy (EDS) line-scan analysis of the carbon steel surface after 24 h of immersion in a 3.5 wt.% NaCl solution containing the protic ionic liquid PIL 02. SEM cross-sectional image indicating the region selected for the line-scan analysis. Corresponding elemental intensity profiles for (A) C, (B) Fe, (C) Cl, (D) O, and (F) N, showing the elemental distribution across the corrosion layer and the steel substrate interface in the presence of the corrosion inhibitor

The formation of this corrosion product occurs only under specific pH and temperature conditions. This study is intended to further investigate the formation of this product within a denser layer of corrosion deposits on steel surfaces (details in figure S8).

Among the elements analyzed in this study, chlorine (Cl) emerges as a key contributor to corrosion processes. This species has the capacity to disrupt the passive protective layer on the metal surface, thereby exposing the underlying substrate and significantly promoting corrosive activity. Moreover, chloride ions tend to form soluble complexes due to their electrostatic affinity with metal cations.

These soluble species facilitate the transport of metal ions away from the surface, thereby accelerating material degradation over time. Energy-dispersive X-ray spectroscopy (EDS) analysis revealed an initial increase in chlorine concentration (%), followed by fluctuations alternating between increases and decreases before stabilizing at an intermediate level across the evaluated distance (see Figure S8 for details).

Similarly, oxygen (O) exhibited a notable increase in concentration, followed by stabilization, a temporary decrease, a subsequent increase, and eventual stabilization before a significant decline. This behavior is likely associated with the sequential formation of various iron oxides and oxyhydroxides. In the system containing PIL 02 (B), the oxide layer appears slightly thicker than that observed for PIL 01 (A), possibly due to differences in the carbon chain length of the compounds.

Lastly, nitrogen (N) initially showed a high concentration, which then decreased, increased again, and ultimately stabilized through the end of the evaluation. This trend is indicative of interactions involving the amine-containing compounds employed as corrosion inhibitors. The formation of a protective film likely results in minor nitrogen deposition on the metal surface, consistent with the observed elemental distribution.

REFERENCES

(1) Pérez, F. R.; Barrero, C. A.; Walker, A. R. H.; García, K. E.; Nomura, K. Effects of Chloride Concentration, Immersion Time and Steel Composition on the Spinel Phase Formation. Mater. Chem. Phys. 2009, 117 (1), 214–223. https://doi.org/10.1016/j.matchemphys.2009.05.045.

(2) Li, S.; Hihara, L. H. In Situ Raman Spectroscopic Study of NaCl Particle-Induced Marine Atmospheric Corrosion of Carbon Steel. J. Electrochem. Soc. 2012, 159 (4), C147–C154. https://doi.org/10.1149/2.013204jes.

(3) Suarez, L.; Coto, R.; Vanden Eynde, X.; Lamberigts, M.; Houbaert, Y. High Temperature Oxidation of Ultra-Low-Carbon Steel. Defect Diffus. Forum 2006, 258–260 (October), 158–163. https://doi.org/10.4028/www.scientific.net/DDF.258-260.158.

(4) dos Santos, N. de O.; Teixeira, L. A.; Zhou, Q.; Burke, G.; C. Campos, L. Fenton Pre-Oxidation of Natural Organic Matter in Drinking Water Treatment through the Application of Iron Nails. Environ. Technol. 2022, 43 (17), 2590–2603. https://doi.org/10.1080/09593330.2021.1890838.

(5) Turney, J. N.; Weiss, D.; Muxworthy, A. R.; Fraser, A. Greigite Formation in Aqueous Solutions: Critical Constraints into the Role of Iron and Sulphur Ratios, PH and Eh, and Temperature Using Reaction Pathway Modelling. Chem. Geol. 2023, 635 (June), 121618. https://doi.org/10.1016/j.chemgeo.2023.121618.

(6) Gnanaprakash, G.; Mahadevan, S.; Jayakumar, T.; Kalyanasundaram, P.; Philip, J.; Raj, B. Effect of Initial PH and Temperature of Iron Salt Solutions on Formation of Magnetite Nanoparticles. Mater. Chem. Phys. 2007, 103 (1), 168–175. https://doi.org/10.1016/j.matchemphys.2007.02.011.

(7) Cornell, R. M.; Giovanoli, R. Effect of Solution Conditions on the Proportion and Morphology of Goethite Formed from Ferrihydrite. Clays Clay Miner. 1985, 33 (5), 424–432. https://doi.org/10.1346/CCMN.1985.0330508.

(8) Furcas, F. E.; Lothenbach, B.; Mundra, S.; Borca, C. N.; Albert, C. C.; Isgor, O. B.; Huthwelker, T.; Angst, U. M. Transformation of 2-Line Ferrihydrite to Goethite at Alkaline PH. Environ. Sci. Technol. 2023, 57 (42), 16097–16108. https://doi.org/10.1021/acs.est.3c05260.

(9) Peretyazhko, T. S.; Ming, D. W.; Rampe, E. B.; Morris, R. V.; Agresti, D. G. Effect of Solution PH and Chloride Concentration on Akaganeite Precipitation: Implications for Akaganeite Formation on Mars. J. Geophys. Res. Planets 2018, 123 (8), 2211–2222. https://doi.org/10.1029/2018JE005630.

(10) Naik, D. L.; Sajid, H. U.; Kiran, R.; Chen, G. Detection of Corrosion-Indicating Oxidation Product Colors in Steel Bridges under Varying Illuminations, Shadows, and Wetting

Conditions. Metals (Basel). 2020, 10 (11), 1439. https://doi.org/10.3390/met10111439.

(11) Nijah Akram; Sajjad Mubin; Ayesha Mehmood Malik; Rimsha Imran; Maryam Jamil. Decoding the Interplay of Lighting and Spatial Dynamics: A Simulation-Based Case Study Analysis across Diverse Building Orientations. Metall. Mater. Eng. 2024, 30 (4), 434–458. https://doi.org/10.63278/10.63278/mme.v31.1.

(12) Shamsa, A.; Barker, R.; Hua, Y.; Barmatov, E.; Hughes, T. L.; Neville, A. The Role of Ca2+ Ions on Ca/Fe Carbonate Products on X65 Carbon Steel in CO2 Corrosion Environments at 80 and 150 °C. Corros. Sci. 2019, 156 (May), 58–70. https://doi.org/10.1016/j.corsci.2019.05.006.

(13) Jevremović, I.; Debeljković, A.; Singer, M.; Achour, M.; Nešić, S.; Miskovic-Stankovic, V. A Mixture of Dicyclohexylamine and Oleylamine as a Corrosion Inhibitor for Mild Steel in NaCl Solution Saturated with CO2 under Both Continual Immersion and Top of the Line Corrosion. J. Serbian Chem. Soc. 2012, 77 (8), 1047–1061. https://doi.org/10.2298/JSC120222058J.

(14) Alcántara, J.; Chico, B.; Simancas, J.; Díaz, I.; de la Fuente, D.; Morcillo, M. An Attempt to Classify the Morphologies Presented by Different Rust Phases Formed during the Exposure of Carbon Steel to Marine Atmospheres. Mater. Charact. 2016, 118, 65–78. https://doi.org/10.1016/j.matchar.2016.04.027.

(15) Santana Rodríguez, J. J.; Santana Hernández, F. J.; González González, J. E. XRD and SEM Studies of the Layer of Corrosion Products for Carbon Steel in Various Different Environments in the Province of Las Palmas (The Canary Islands, Spain). Corros. Sci. 2002, 44 (11), 2425–2438. https://doi.org/10.1016/S0010-938X(02)00047-1.

(16) Yousif, Q. A.; Al-Zhara, A. A. Electrochemical Methods, Sem-Eds and Afm Studies for Assessing Corrosion Inhibition of Carbon Steel in Acidic Media. ARPN J. Eng. Appl. Sci. 2016, 11 (21), 12619–12630.

(17) Castaño, J. G.; Botero, C. A.; Restrepo, A. H.; Agudelo, E. A.; Correa, E.; Echeverría, F. Atmospheric Corrosion of Carbon Steel in Colombia. Corros. Sci. 2010, 52 (1), 216–223. https://doi.org/10.1016/j.corsci.2009.09.006.

(18) Verma, C.; Ebenso, E. E.; Quraishi, M. A.; Hussain, C. M. Recent Developments in Sustainable Corrosion Inhibitors: Design, Performance and Industrial Scale Applications. Mater. Adv. 2021, 2 (12), 3806–3850. https://doi.org/10.1039/D0MA00681E.

(19) Garcia, S. J.; Markley, T. A.; Mol, J. M. C.; Hughes, A. E. Unravelling the Corrosion Inhibition Mechanisms of Bi-Functional Inhibitors by EIS and SEM-EDS. Corros. Sci. 2013, 69, 346–358. https://doi.org/10.1016/j.corsci.2012.12.018.

(20) Rosliza, R.; Izman, S. SEM-EDS Characterization of Natural Products on Corrosion Inhibition of Al-Mg-Si Alloy. Prot. Met. Phys. Chem. Surfaces 2011, 47 (3), 395–401. https://doi.org/10.1134/S2070205111030129.
